# A Comprehensive Review of Numerical and Machine Learning Approaches for Predicting Concrete Properties: From Fresh to Long-Term

**DOI:** 10.3390/ma18153718

**Published:** 2025-08-07

**Authors:** Nilam Adsul, Yongho Choi, Su-Tae Kang

**Affiliations:** 1 Department of Civil Engineering, Daegu University, Gyeongsan 38453, Republic of Korea; adsulnil@daegu.ac.kr; 2 Faculty of Department of Computer & Information Engineering (Cyber Security), Daegu University, Gyeongsan 38453, Republic of Korea; yongho_choi@daegu.ac.kr; 3 Faculty of Department of Architecture Engineering, Daegu University, Gyeongsan 38453, Republic of Korea

**Keywords:** modelling techniques, internal curing, cementitious materials, concrete properties, prediction

## Abstract

The growing demand for innovation and the use of diverse materials in cementitious composites necessitate predictive models that account for material variability. Numerical, code-based, and machine learning (ML) models have been developed to predict various concrete properties. However, their accuracy is significantly influenced by factors such as mix design, composition, intrinsic properties, and external conditions. Developing robust models that integrate these variables is essential for improving predictive accuracy and optimizing material performance. This paper presents a comprehensive review of numerical, code-based, and ML modelling techniques for predicting both fresh and long-term concrete properties. Since both numerical and ML models rely on experimental data—either to determine coefficients in numerical approaches or to train ML models—data gathering, preprocessing, and handling are crucial for model performance. Previous studies indicated that data variability significantly impacts accuracy, emphasizing the importance of effective preprocessing. While larger datasets generally improve reliability, some models achieve high accuracy even with very limited data. This review not only demonstrates the superior performance of ML models over traditional numerical approaches but also highlights the relative effectiveness of different ML algorithms based on reported accuracy metrics. ML-based approaches, including both ensemble and non-ensemble models, have exhibited strong predictive capabilities across a wide range of concrete property categories. In contrast, traditional numerical models often yield lower accuracy, although modified versions that incorporate additional parameters have shown improved performance. Furthermore, the integration of optimization algorithms and interpretability tools enhances both predictive reliability and model transparency—critical aspects that are often overlooked.

## 1. Introduction

Infrastructure such as roads, bridges, tunnels, dams, power plants, ports, airports, seawalls, and water treatment facilities heavily relies on concrete, much like building foundations and, in many cases, entire structures [1,2]. Concrete’s widespread use in construction can be attributed to its beneficial properties, including versatility, compressive strength (CS), elastic modulus (EM), durability, hardness, porosity, density, and resistance to fire and heat. Among these properties, CS and EM are considered the most crucial in structural design, as they play a key role in ensuring the safety and longevity of concrete structures [3,4,5,6]. However, achieving exceptional concrete properties requires careful design and extensive laboratory testing, making the process highly time-consuming, labor-intensive, costly, and environmentally damaging. Additionally, the results are influenced by variations in test conditions and operator expertise [2,7,8,9,10,11]. Furthermore, attaining high mechanical performance in concrete design is difficult because conventional production methods struggle to create an ideal microstructure, which is key to preventing concrete failure. These conventional methods include optimizing mix proportions, adjusting aggregate particle size distribution, and refining experimental procedures [12,13,14,15,16,17,18].

As a result, many researchers have been and continue to be engaged in developing prediction models that accurately predict concrete properties while minimizing the need for extensive experiments, as well as reducing costs, time, and labor. Numerous numerical models—including regression, empirical, semi-empirical, and analytical approaches, have been proposed to achieve more constrained ranges of material consumption for obtaining targeted concrete properties [19,20]. Balshin’s model is recognized as one of the most widely recognized models for predicting the CS of aerated concrete. Among various pore characteristics—such as porosity, pore size, and shape—the model specifically identifies porosity as the key parameter in its predictions [21]. Similarly, Youssef et al. (2017) [22] utilized two other models alongside the Balshin model, namely Schiller and Ryshkewitch, to predict the mechanical strength of porous solids based on their porosity. This approach provides a theoretical framework for evaluating the compressive strength of foam concrete [22,23,24]. The Balshin-type power laws effectively predicted the Young’s modulus and compressive strength, while the Ryshkewitch-type law provided an acceptable prediction of the relative compressive strength of foam concrete based on its macro porosity. Additionally, the Schiller-type equation offered a reliable estimation of the relative flexural tensile strength [22].

Kiang (2006) [25] employed multiple models to predict creep and shrinkage and conducted experimental testing on concrete specimens with characteristic compressive strengths of 20, 30, and 40 MPa. The test results were compared with several prediction models, including Eurocode 2 (EC2) EN 1992-1-1 (2004) [26], ACI 209 [27], CEB-FIP 1990 [28,29], B3 [30,31], GL 2000 [32], and Australian Standard AS3600 [33]. The AS3600 model provided the most accurate creep predictions, while the B3 model was most effective for shrinkage. However, the CEB-FIP 1990 model was preferred over AS 3600 for creep prediction, as AS3600 relies on graphical interpretation, raising concerns about accuracy. To improve predictions, modification factors were proposed for both the CEB-FIP 1990 and B3 models. Similarly, based on conversion formulas from various standards (AS3600 [33], EC2 EN 1992-1-1 (2004) [26], ACI 318-14 [34], CEB-FIP 2010 [35], and JTG/T F30-2014 [36]) and research studies, Xie et al. (2020) [37] proposed and verified equations relating the splitting tensile strength (TS), CS, and flexural strength (FS) of superabsorbent polymer (SAP) concrete using experimental data. Comparative analysis confirmed the high applicability of these equations, making them suitable for practical use in SAP concrete applications.

Ali and Mostafa (2014) [38] developed a mathematical model to predict the CS of chemically activated cement mortar with high phosphorous slag content. Using curve fitting and regression analysis, the model incorporates curing time and water-to-cement (w/c) ratio as input variables. The logarithmic model features two parameters: α, a constant, and β, which depends solely on the w/c ratio. The model is theoretically robust, user-friendly, and capable of accurately predicting CS across different Blaine fineness levels and curing durations. Its average error does not exceed 3%, confirming its reliability.

However, traditional numerical models used to predict concrete properties face several notable limitations. This is primarily due to the complex, highly nonlinear, and inelastic behavior of concrete, which is difficult to capture accurately using simplified constitutive models. These conventional approaches often depend on predefined equations and assumptions, which restrict their accuracy and applicability—especially when addressing complex structural behaviors or varied environmental exposure conditions [39,40,41].

As a result, the application of artificial intelligence (AI) has gained significant traction in the construction industry. Machine learning (ML) and deep learning (DL) techniques have shown great promise for predicting the properties of concrete. ML, a subset of AI, uses algorithms that learn from data to perform intelligent tasks, while DL—a more advanced form of ML—employs multi-layered neural networks to model intricate patterns and relationships [11,42,43]. Unlike traditional models that rely on solving fundamental equations, ML models learn directly from experimental or computational data. They are particularly effective at identifying complex, multidimensional patterns within large datasets, often uncovering insights that are inaccessible through traditional analytical methods. As such, ML offers a powerful approach for exploring extensive material design spaces, revealing hidden trends, and predicting behavior in scenarios beyond current experimental or computational capabilities [44,45,46].

In cementitious materials, ML-based data-driven approaches enable a deeper understanding and optimization of the complex interactions among raw materials, supplementary cementitious materials (SCMs), admixtures, and environmental conditions. These interactions are often highly nonlinear and involve poorly understood coupling effects, making them difficult to model accurately using traditional methods—particularly in relation to hydration kinetics, mechanical performance, and durability [47,48].

ML models are generally categorized into non-ensemble and ensemble types. Non-ensemble models rely on a single predictive approach without combining multiple models. These include Multiple Linear Regression (MLR), Multiple Nonlinear Regression (MNLR), Support Vector Regression (SVR), Gene Expression Programming (GEP), Artificial Neural Networks (ANNs), and the Adaptive Neuro-Fuzzy Inference System (ANFIS) [2,3,11,49,50]. In contrast, ensemble ML enhances predictive performance by integrating multiple models. Used for both regression and classification tasks, ensemble techniques fall into three main categories: Bagging, Boosting, and Stacking. These methods help reduce bias and variance, improve model diversity, and enhance the interpretability of regression predictions [2]. The decision to use a non-ensemble or ensemble model depends on the specific problem, dataset characteristics, and performance needs. Non-ensemble models are more straightforward and require less computational power, making them ideal for tasks where interpretability and efficiency are essential [51].

Non-ensemble modelling has been widely utilized by researchers. Njoroge et al. (2024) [52] used neural networks for more robust and adaptable predictions, utilizing data from laboratory experiments. Wu et al. (2024) [19] employed four key training algorithms to optimize ANN models. Resilient Backpropagation (trainrp) dynamically adjusts step sizes for fast convergence, making it ideal for large-scale problems. Levenberg–Marquardt (trainlm) combines gradient descent and Newton’s methods, offering rapid convergence for small- to medium-sized problems. Bayesian Regularization (trainbr) enhances generalization and prevents overfitting, making it effective for complex problems with limited data. Scaled Conjugate Gradient (trainscg) is a memory-efficient variant that ensures fast convergence and is suitable for large datasets. Many studies have used feedforward neural networks, with the most used type being the Multilayer Perceptron (MLP), which consists of an input layer, one or more hidden layers, and an output layer [10,52,53,54,55,56,57]. Keshavarz and Torkian (2017) [58] used two modelling techniques, ANN and ANFIS, to predict the CS of concrete based on parameters such as cement, w/c ratio, gravel, sand, and micro silica. Both models performed well, with ANN achieving an R^2^ of 0.942 and ANFIS an R^2^ of 0.923. Paudel et al. (2023) [59] applied MLR and SVR to predict the CS of concrete containing fly ash (FA) and compared the accuracy of different models. The MLR model exhibited lower accuracy, with an adjusted coefficient of determination (R^2^) of 0.49, a high error distribution (Mean Absolute Error (MAE) = 7.68 MPa, Mean Square Error (MSE) = 88.43 MPa^2^), and limited suitability for predicting nonlinear outcomes. In contrast, the SVR model showed improved performance with an adjusted R^2^ of 0.84, which was further enhanced through hyperparameter tuning techniques such as random search, grid search, and Bayesian optimization. Before tuning, the SVR model had R^2^ = 0.55, MAE = 7.20 MPa, and MSE = 81.99 MPa^2^, which improved to R^2^ = 0.85, MAE = 3.76 MPa, and MSE = 27.06 MPa^2^ after tuning, demonstrating reduced error and enhanced model accuracy.

In ensemble modelling, the Bagging Regressor (Bootstrap Aggregating) is used for regression tasks. It trains multiple instances of the same algorithm on different bootstrap samples—random subsets of the original dataset with replacement. The final prediction is obtained by averaging the outputs of these models, reducing variance and improving robustness. This approach enhances predictive accuracy while minimizing overfitting [51]. Boosting improves prediction accuracy by combining multiple weak models into a strong one. Instead of relying on a single highly accurate rule, boosting iteratively applies a base learning algorithm to different subsets or weighted distributions of training data. Each iteration produces a weak prediction model, and the boosting algorithm integrates these models to form a more accurate final prediction. This method enhances performance by refining weak rules and reducing errors through repeated learning cycles [60]. Stacking, on the other hand, follows a two-layer structure consisting of a base model and a meta-model [2]. It enhances prediction accuracy and generalizability by combining multiple model predictions into a single meta-learner, leading to stronger predictive performance [61]. Stacking is further classified into two types: the Integrated Stacking Model (ISM), where a neural network serves as the meta-learner, and the Separate Stacking Model (SSM), which employs a different type of meta-learner. In the study by Barkhordari et al. (2024) [62], the Decision Tree regressor (DTR), Gradient Boosting regressor (GBR), Random Forest Regressor (RFR), AdaBoost regressor (ABR), and Bagging regressor (BR) algorithms were used as meta-learners in the SSM.

Bagging and boosting are widely used techniques, particularly for handling data imbalance. While both methods are effective, boosting demonstrates superior performance when the imbalanced data is oversampled [63]. Common ensemble models include Adaptive Boosting (AdaBoost), Random Forest (RF), Extreme Gradient Boosting (XGB), and Categorical Gradient Boosting (CatBoost) [3]. XGB is an advanced ML algorithm based on Gradient Boosting Decision Trees (GBDT), while RF is a bagging technique inspired by ensemble learning, consisting of multiple decision trees operating in parallel as meta-learners [12,64]. The K-Nearest Neighbors (KNN) algorithm predicts outcomes by considering the most frequently occurring response among the K closest data points to the test sample [65].

Taiwo et al. (2024) [66] proposed ensemble techniques leveraging the strengths of multiple base models, such as XGB and CatBoost. The performance of these ensemble models was compared against eight standalone algorithms to assess their effectiveness in predicting the CS of high-performance concrete (HPC). The standalone algorithms included Logistic Regression (LR), SVM, RF, XGB, AdaBoost, CatBoost, Light Gradient Boosting Machine (LightGBM), and MLP. Nguyen et al. (2024) [67] employed a data-driven approach to predict and evaluate the mechanical properties of concrete made with recycled concrete aggregate (RCA), focusing on CS and EM. Gradient Boosting and Categorical Boosting achieved the highest accuracy for CS, with R^2^ values of 0.9112 and 0.9175, Root Mean Square Error (RMSE) values of 5.3464 MPa and 5.1520 MPa, and MAE values of 4.0845 MPa and 3.7567 MPa, respectively. For EM prediction, LightGBM and CatBoost performed best, with R^2^ values of 0.8775 and 0.9300, RMSE values of 2.3560 GPa and 2.3560 MPa, and MAE values of 1.8330 GPa and 1.2589 MPa. Song et al. (2021) [10] deployed ML based methodologies, including ANN, RF, Decision Tree (DT), and Gradient Boosting Networks.

In the stacking ensemble technique, multiple base models—including DT, SVM, RF, KNN, XGB, and Bagging—were selected. Their optimal hyperparameters were determined using grid search with five-fold cross-validation. The prediction outputs from these models were then analyzed using an optimal meta-model. Additionally, these base models were tested as meta-models, and their performances were compared to identify the best one. The final meta-model was trained using the base models’ predictions as features. Results showed that RF performed the best, achieving an R^2^ value of 0.88 and a MSE of 22.06. Due to its superior performance, RF was chosen as the meta-model for further analysis in the stacking ensemble regression model [2]. Duan (2024) [65] incorporated two meta-heuristic methods, the Fire Hawk optimizer (FHO) and Runge–Kutta optimization (RUK) to enhance model accuracy with KNN analysis. The research results reveal three separate models: KNFH, KNRK, and a single KNN model, each providing valuable insights for precise CS prediction.

**Figure 1:**
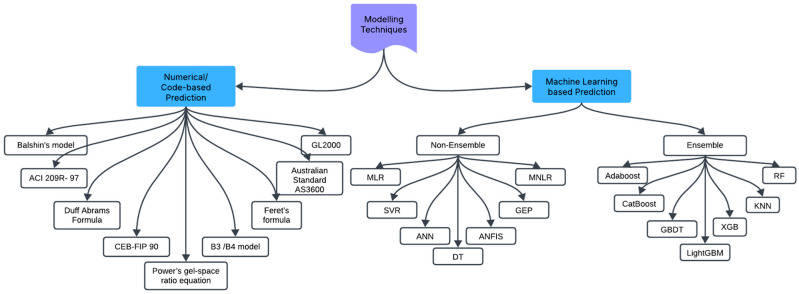
Prediction modelling techniques.

The graphical illustration of a few types of prediction modelling techniques is shown in Figure 1. Various prediction modelling techniques have been studied by researchers. To provide a comprehensive overview, we conducted a detailed review of different numerical, code-based, and ML modelling techniques used for predicting concrete properties. This review also covers data processing, verification, and model validation techniques. It aims to assist researchers in identifying the most effective modelling approach for predicting specific concrete properties.

## 2. Methodology, Data Handling, and Data Processing Techniques

**Figure 2:**
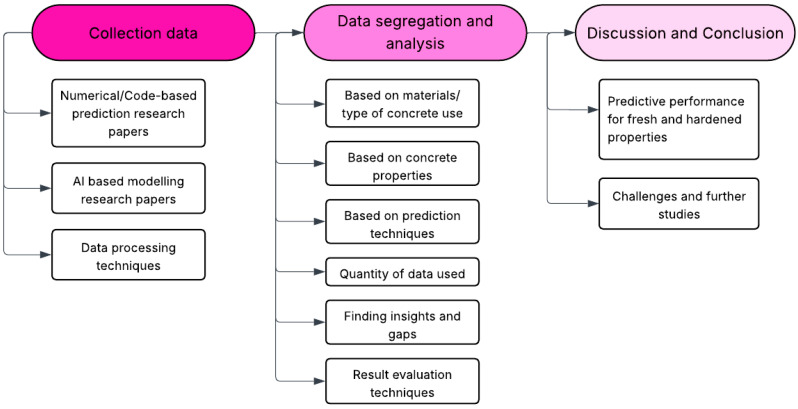
Methodological approach adopted for the review.

The methodological approach used for this review was illustrated in Figure 2 and consisted of three main stages. The first stage involved the collection of relevant research articles related to numerical, code-based, and AI-based prediction modeling, as well as those focused on data processing and handling. In the second stage, the collected articles were categorized based on the type of material/concrete studied—such as cement paste, high-performance concrete (HPC), high-strength concrete (HSC), alkali-activated concrete, and concrete with or without internal curing materials. Additionally, the categorization included the specific concrete properties targeted for prediction and the modeling techniques employed. This was followed by data analysis, which considered factors such as the materials used, the properties evaluated, the modeling approaches applied, the quantity of data involved, identified research insights and gaps, and the methods used for evaluating results. Finally, the review discussed the predictive performance for various fresh and hardened concrete properties, highlighted existing challenges, and outlined directions for future research, concluding with key findings.

Further, data gathering and processing are crucial steps in every prediction modelling technique. Researchers have utilized a wide range of datasets to enhance model accuracy. For instance, Paudel et al. (2023) [59] used a dataset of 633 experimental results (CS ranging from 6.27 MPa to 79.99 MPa) collected from previously published literature. The key input variables included cement (C), fine aggregate (FA), coarse aggregate (CA), fly ash, water content (W), superplasticizer percentage (SP), and curing day. Another dataset, consisting of 1030 test samples, incorporated eight input variables: C, blast furnace slag (BSF), fly ash, W, SP, CA, FA, and age. The dataset was divided into 60% for training, 20% for validation, and 20% for testing. The training set was used to develop the prediction model, the validation set helped fine-tune hyperparameters and prevent overfitting, and the testing set assessed the model’s performance to ensure accuracy and reliability [68]. Wu et al. (2024) [19] utilized a dataset containing 271 sets and 3252 experimental data points. Ziyad Sami et al. (2023) [69] observed that in ML, increasing the number of input variables generally improved accuracy. However, in some cases—such as exponential Gaussian Process Regression (GPR)—the model effectively predicted CS even with just five input variables during testing. For TS prediction, the number of input variables had minimal influence on model accuracy in both training and testing phases. Overall, model accuracy largely depends on data quality and the selected input variables. Similarly, a major limitation of ML models in predicting concrete properties is the lack of high-quality data, which can impact model accuracy. Concrete characteristics are influenced by multiple factors, including mix design, curing conditions, and environmental variables, leading to significant variability. This inherent fluctuation in concrete properties can reduce the precision and reliability of ML models [70].

**Figure 3:**
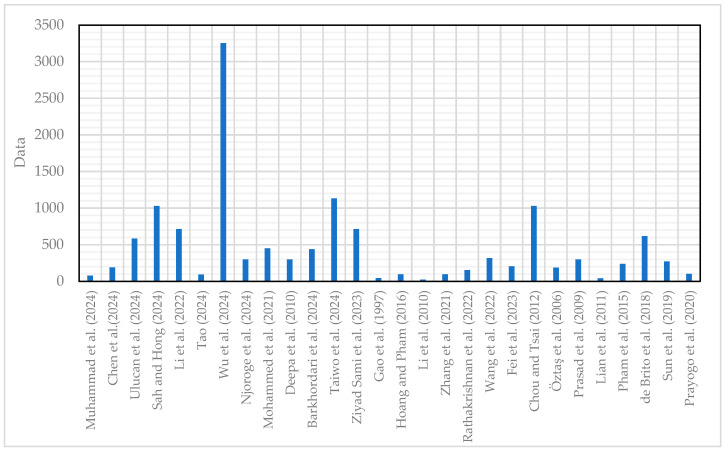
Range of dataset used for modelling [4,7,8,9,11,12,19,52,53,55,62,66,69,71,74,75,76,77,78,79,80,81,82,83,84,85,86,87].

While many researchers have used small datasets with fewer than 100 data points [4,12,56,71,72,73], they still achieved good prediction results. Hoang and Pham (2016) [74] collected 95 concrete mix data points from the Song Bung 2 Hydroelectric Dam project. Li et al. (2010) [75] used data from 23 concrete mixes. In another study, a total of 96 sets of data were used to form a database to predict FS [76]. Table 1 and Figure 3 present details of the modelling data and dataset range used in previous studies for predicting concrete properties.

In ML, a portion of the dataset is allocated for training and testing, with the training set typically being larger than the testing set. In most cases, an 80/20 split is used for modelling. Unlike Gradient Boosting Trees (GBT), RF inherently incorporates a 63/37 random partition within the bootstrap sampling process, eliminating the need to split the dataset into 75/25 training and testing subsets [88].

In a study by Sah and Hong (2024) [9], several data preprocessing techniques were applied, including outlier detection, handling missing values, and feature scaling. These steps ensured data usability and enhanced result accuracy. The study utilized eight key variables—C, BFS, fly ash, W, SP, CA, FA, and age—to predict the CS of concrete, all of which are essential in concrete manufacturing. When certain components were unavailable in the production process, null values were incorporated into the prediction model [9], or missing data in the dataset were handled by replacing them with mean values during analysis [89]. Additionally, data normalization plays a crucial role in preprocessing by standardizing input and output values within a consistent range, thereby improving model performance. Several normalization techniques are commonly used, including min–max scaling, which rescales values within a fixed range (e.g., [0, 1] or [−1, 1]); decimal scaling, which adjusts values by dividing them by powers of 10; and Z-score normalization, which standardizes data by subtracting the mean and dividing by the standard deviation (SD). These techniques help eliminate numerical discrepancies, reduce biases from disproportionately large variables, and enhance the stability and efficiency of ML model training [12]. Similarly, Chen et al. (2024) [7] normalized the input and output values to the range of [−1, 1] using Equation (1), ensuring that all data features were on a similar scale—an essential step for algorithms that depend on distance metrics or weight adjustments during optimization. This process accelerates convergence during training, enhances model stability, and reduces sensitivity to absolute magnitudes, allowing the model to focus on the relative importance of features.

(1) $$y=\frac{\left(y_{max}-y_{min})(x_{max}-x_{min}\right)}{x_{max}-x_{min}}+y_{min}$$

where *x* and *y* represent the input and output values, respectively. The target range for normalization is denoted as $(y_{max},y_{min}),$ while ${(x}_{max},x_{min})$ represents the range of input values being normalized.

After data preprocessing, a data encoding procedure was applied. During this process, the strength reduction percentage (output) was categorized into three groups and assigned encoded values from 0 to 2: (1) 0 for cases where strength reduction was negative or SAP concrete exhibited higher strength than normal concrete, (2) 1 for strength reductions between 0% and 15%, and (3) 2 for reductions between 15% and 35%. To address data imbalance in the classification process for predicting strength reduction, Synthetic Minority Oversampling Technique (SMOTE) was applied. The largest dataset belonged to classification 1, which led to an inaccurate prediction model. To correct this, SMOTE generated synthetic samples, ensuring a balanced dataset across all classifications. This oversampling method increases the number of minority class instances by creating synthetic duplicates rather than simple replication, stabilizing the class distribution. SMOTE was applied only during training, while the original experimental dataset was preserved for testing [89].

**Figure 4:**
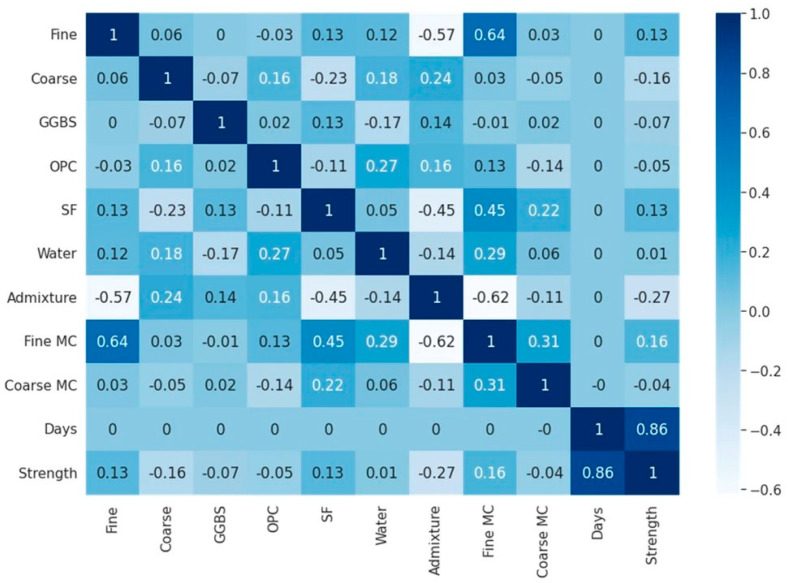
Pearson’s correlation heatmap [77].

Furthermore, the Pearson Correlation Coefficient (PCC) measures the strength and direction of the linear relationship between input and output values. It is derived using the covariance approach, which is effective for assessing the connection between variables of interest. Its value ranges from −1 to 1, as shown in Figure 4; a positive value indicates a positive correlation, while a negative value signifies a negative correlation [7,77].

Additionally, K-fold cross-validation is a widely used method for evaluating the generalization and reliability of ML models. It involves splitting the dataset into K equal-sized subsets (folds). The model is then trained and evaluated K times, with each iteration using one subset for testing while the remaining K − 1 subsets are used for training. This iterative process reduces the impact of data partitioning, providing a more reliable and unbiased performance evaluation [7,12,78,88]. A typical implementation of K-fold cross-validation uses ten folds, where nine folds are used for training and one-fold is reserved for testing in each iteration. This ensures that every subset serves as the test set once. By averaging the results across all iterations, this method captures data variability while maintaining computational efficiency. Systematically training and testing the model enhances reliability and reduces the risk of overlooking crucial data [3].

In contrast, simple cross-validation evaluates the algorithm using the dataset only once, making the evaluation index highly dependent on the data split ratio and limiting its effectiveness. K-fold cross-validation addresses this by improving data utilization and optimizing parameter tuning. Fei et al. (2023) [79] employed five-fold cross-validation, dividing the dataset into five equal subsets. Each subset took turns as the validation set, while the remaining samples formed the training set. This process was repeated five times, and the final evaluation index was obtained by averaging the results of all five iterations, ensuring a more reliable and robust model evaluation. Tao (2024) [12] used ten-fold cross-validation, a preferred choice for small datasets, which minimizes MSE, bias, and standard deviation, leading to more accurate performance estimates.

**Table 1:** Modelling data details used in previous studies.

Ref.	Type of Concrete	Input	Output	Model Evaluation Parameters	Dataset/Range	Model Interpretation and Modelling Technique
[4]	HPC	slump, w/b, W, FA, s/a, SF, and SP	28D CS	R, PCC and MAPE, MSE, MAE	77	Generalized Regression Neural Network, Nonlinear Auto Regressive with exogenous inputs (NARX neural network), and RF, Radial Basis Function Neural Network
[7]	Concrete	C, fly ash, mineral powder content, FA, CA, W, and SP	28D Cube CS	F1-score for classification tasks, MSE, RMSE, and MAE	189	Backpropagation Neural Network (BPNN), SVM, and RF, PSO algorithm
[9]	Concrete	C, BFS, Fly ash, W, SP, CA, FA, age	28D CS	MAD, RMSE, CC, MAPE	1030	ANN, MLR, SVM, and a regression tree
[10]	HPC	C, FA, W, SP, CA, fly ash, age	CS	MAE, MSE, RMSE, and RMSLE	471	MLP, DT, RF and GBR
[12]	Nano-modified concrete	w/c, carbon nanotubes, nano-silica, nano-clay, nano-aluminum, C, CA, and FA	Uniaxial CS	R^2^, MAPE, RMSE, RSR, and NMBE	94	BPNN, RF, XGB, and HEStack
[19]	Pervious concrete	C, CA, minimum Agg. Dmin and maximum Agg. Dmax diameter of CA, CA type, sand, w/c, content of additions, content of additives, porosity	Permeability and CS	R^2^	3252	ANN (MLP)
[55]	HSC	w/b, W, FA, fly ash replacement ratio, SF replacement ratio and SP	28D CS	RMSE, MAE, R	300	Multilayer Perceptron (MLP), M5P Tree models and LR
[62]	Concrete	w/c, w/b, a/c, C, SF, Fly ash, BFS, MK, Filler, SP, SAP%, SAP size, SAP water uptake (%), time	AS	RMSE, R^2^, Overall Index of model performance, MAE, and 95% Uncertainty	437	Simple Average Ensemble, Snapshot Ensemble, and Stacked Generalization, integrated stacking model (ISM), SHAP, KNN, RF, Gradient Boosting (GB), and XGB
[66]	HPC	C, BFS, Fly ash, W, SP, CA, FA, age of testing	CS	R^2^, RMSE, MAE, MAPE, RRSE, RMSLE, R, KGE, NSE	1133	ELimination Et Choix Traduisant la REalité (ELECTRE), Recursive Feature Elimination (RFE), SHAP, logistic regression, SVM, RF, XGB, AdaBoost, CatBoost, LightGBM, and MLP
[70]	Normal concrete	C, BS, fly ash, water, SP, CA, FA, and age	28D CS	R^2^, RMSE, MAE	1030	RFR and CatBoost, fivefold cross-validation technique
[71]	Aluminate cement pastes and concrete	Amount of SAP, Size of SAP (mesh), and C/W	CS, modulus of elasticity, and split TS	Standard deviation (SD)	45	Box–Behnken design
[72]	Cellular concrete	Density of fresh concrete, w/c, s/c, vol./weight of C, W, and air, degree of hydration	28D CS	R^2^	96	Duff Abrams Formula, Feret’s formula, Powers’ modified gel/space ratio formula
[80]	HPC	C, BFS, Fly ash, W, SP, CA, FA, Age of testing	28D CS	MAPE, RMSE	1030	Linear regression (LR), ANN, SVR
[81]	HSC	w/b, W, FA, fly ash replacement ratio, air-entraining agent ratio, SF replacement ratio and SP	CS and slump	RMS, SSE, R^2^, MAPE	132	ANN (BPNN)
[82]	SCC and HPC	C, w/c, w/b, w/p (P = C + FA + MS), FA/P, CA/P, HRWR/P, VMA/P, FA/B, MS/B	Slump and 28D CS	R^2^, Error	300	ANN (Matlab Neural Network Toolbox and Alyuda NeuroIntelligence (2001))
[83]	Porous concrete	Cement to aggregate, specific gravity of binder, aggregate density, sample density, total porosity, CS	Relation between porosity and CS	R^2^	Approx. 42	Griffith’s fracture theory, Multiple linear regression run by least square method
[90]	Cement paste with/without GGBS	RH, w/c, volume fraction of the aggregates, shrinkage of the paste, correlation parameter controlled by aggregate restraining effects	Drying (DS) and autogenous shrinkage (AS)	R^2^	Approx. 40	HYMOSTRUC model, Pickett model
[84]	HSC	C, sand, small CA, medium CA, W, and SP	3-, 7-, and 28-day CS	RMSE, MAPE, R^2^	239	Least Squares Support Vector Regression Firefly Algorithm (FFA)
[85]	concrete	C and NA	28D CS	R^2^	618	Abrams, Slater and ACI models, two modified models (Bolomey and Feret)
[86]	Pervious concrete	w/c, a/c, and aggregate size	Permeability coefficient and 28D unconfined CS (UCS)	RMSE, R	270	Evolved support vector regression (ESVR) tuned by beetle antennae search (BAS)
[87]	Concrete	C, BFS, fly ash, W, SP, CA, FA	Slump and 28D CS	R, MAE	103	LR analysis, classification and regression tree analysis, Chi-squared automatic interaction detection, ANN, and SVM

Note: w/b = water-to-binder ratio, BFS = slag or blast furnace slag, NA = natural aggregates, P = powder = cement + supplementary cementitious materials (SCMs), B = binder = cement + SCMs, MK = metakaolin, HRWR = high range water reducer, VMA = viscosity modify admixture, MS = micro silica, a/c = aggregate-to-cement ratio, s/c = sand/cement ratio, SCC = high strength self-compacting concrete, R = correlation coefficient, RMSE = root mean square error, MABE = mean absolute bias error, RRMSE = relative RMSE, MAPE = relative MABE, NMBE = normalized mean bias error, Ef = Coefficient of efficiency, SI = scatter index, PCC = Pearson Correlation Coefficient, RSR = standard deviation of observations, KGE = Kling–Gupta efficiency, NSE = Nash–Sutcliffe efficiency, RMSLE = root mean square log error.

## 3. Modelling Techniques

There are various properties of cementitious materials that help determine their intended use, particularly in fresh and hardened concrete. Fresh concrete properties include workability, fundamental rheological characteristics, thixotropy, slump loss, setting time, bleeding, segregation, and more. For hardened concrete, key properties encompass compressive strength, along with mechanical and physical attributes such as TS, elasticity, shrinkage, creep, cracking resistance, and electrical, thermal, and transport properties [91]. Among these, a few widely tested and modelled properties for predictive analysis are discussed below.

### 3.1. Fresh Properties

Various researchers have employed a range of mathematical and ML techniques to predict the fresh concrete properties. Among these methods, ANNs have been the most widely used [75,81,92,93]. For instance, Li et al. (2010) [75] developed an ANN model to predict the workability of SCC based on parameters such as slump, slump flow, and V-test results. Using laboratory experiments data from 23 concrete mixes, the model was trained with six input parameters including C, fly ash, BFS, SP, sand ratio, and w/b ratio. Three ANN models (ANN-1, ANN-2, and ANN-3) with 15, 11, and 5 neurons in their hidden layers, respectively, were evaluated. Of these, ANN-2 provided the most accurate predictions for SCC workability. Singh et al. (2021) [92] also employed an ANN to determine the optimal equation for predicting workability and CS for concrete with fly ash and slag. Agarwal and Sharma (2010) [93] utilized a multilayered feedforward BPNN, based on the Levenberg–Marquardt learning rule, and trained with eight input parameters: C, fly ash, sand, CA (10 mm and 20 mm), W, SP, and w/b ratio. To assess the model’s accuracy, it was further tested with 40 additional mix designs from another batching plant. The model performed exceptionally well on data from the first batching plant, achieving an MSE of 0.0012 and an R^2^ of 0.9985, confirming its reliability. However, when tested with data from the second batching plant, performance declined, with an MSE of 0.0579 and an R^2^ of 0.9185, indicating that the model’s accuracy is affected by variations in cement type, admixtures, and mix proportions. This highlights the importance of consistency in input data for optimal performance. Bai et al. (2003) [94] developed ANN models to predict the workability of concrete incorporating MK and fly ash, showing that the models were highly reliable in predicting slump, compaction factor, and Vebe time for various PC–FA–MK compositions. The models yielded R values ranging from 0.98 to 1 for slump and compaction factor, and from 0.92 to 0.99 for Vebe time.

Azimi-Pour et al. (2020) [95] utilized SVM models with various kernel functions—linear, polynomial, radial basis function (RBF), and sigmoid—to predict the fresh properties of high-volume fly ash self-compacting concrete (HVF-SCC), addressing the challenge of limited data for such mixtures. The study demonstrated the potential of SVM models trained with various volume fly ash SCC (VVF-SCC) data to predict both fresh properties (slump flow, V-funnel, U-box, L-box ratios) and CS for both low-volume (LVF-SCC) and HVF-SCC mixtures. The models showed improved accuracy when using normalized input vectors, such as w/c, W/P, W/B, FA/P, CA/P, HRWR/P, VMA/P, fly ash/B, and MS/B —compared to using the amount of each component. Additionally, optimizing kernel function parameters through grid search further enhanced prediction performance. The RBF kernel outperformed the other kernels in predicting fresh properties due to its ability to handle nonlinear data. When applied to HVF-SCC experimental results, the SVM model with the RBF kernel demonstrated good accuracy, with R^2^ values of 0.9117 for slump, 0.9295 for V-funnel, 0.9484 for U-box, and 0.9372 for L-box, outperforming other existing models. Overall, this research validated the effectiveness of SVM modelling in predicting SCC properties, particularly when using optimized kernel parameters and appropriate input vectors.

**Figure 5:**
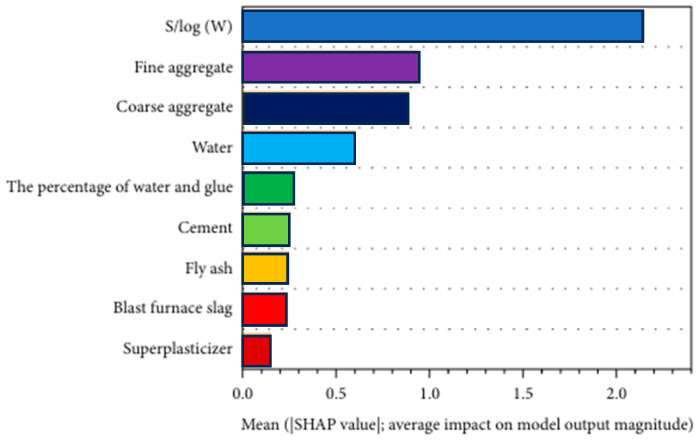
Mean absolute SHAP values of XGB model [96].

Advanced concrete workability prediction models, including ensemble learning models such as RF, XGB, AdaBoost, and Gradient Boosted Regression Trees (GBRTs) were explored. The input parameters include C, BFS, fly ash, W, SP, CA, FA, percentage of W and glue, and S/log(W). XGB outperformed the other models, achieving the highest R^2^ value of 0.962 and the lowest MAPE, MAE, and RMSE values, demonstrating superior predictive capabilities. Additionally, SHAP analysis was used to identify key factors influencing concrete performance as shown in Figure 5, confirming the effectiveness of XGB in evaluating individual and combined variable impacts [96]. SHAP is widely used due to its ability to capture the influence of all features for each sample while indicating whether their impact is positive or negative. SHAP calculates Shapley values based on cooperative game theory principles, where features act as participants in a coalition. These values quantify the contribution of each feature to the model’s predictions, offering a detailed and transparent interpretation of results [3,79]. PDP analysis is another valuable technique for understanding how individual or combined features influence an ML model’s prediction. It helps determine whether the relationship between an input variable and the output is linear or complex [88,97].

Further, a Least Squares Support Vector Regression (LS-SVR) model was employed to predict concrete slump (CSP) by mapping mix components to slump values. To optimize the model’s performance, a grid search was conducted to identify the most suitable hyperparameters (regularization and kernel parameters). The model considered only six conditioning factors (C, natural sand, crushed sand, CA, W, S), excluding parameters such as the type, size, absorption, and water content of the FA and CA, which could enhance prediction accuracy. Experimental results confirm the model’s high accuracy, achieving an average MAPE below 3% through tenfold cross-validation, demonstrating its reliability in modelling the nonlinear behavior of concrete slump. However, limitations exist. The MLR model performed poorly in the testing phase, with an RMSE of 0.28, a MAPE of 12.08%, and an R^2^ of 0.28. This result indicates that a linear model is insufficient to accurately capture the behavior of concrete slump. In contrast, the ANN and CSP-LSSVR models demonstrated significantly better performance, both achieving R^2^ values above 0.90 [74].

Multivariate regression analysis (MRA) was used to model the properties of fresh concrete, such as slump, air content, and density. The study found that the w/c ratio, sand/C ratio, and amount of Air-Entraining Agent (AEA) were key factors affecting slump, while AEA had the most significant effect on air content. For density, the MRA model performed exceptionally well with an R^2^ of 99.1%. The study concluded that while slump prediction remains less reliable due to variations in mix proportions, the MRA model successfully predicted air content and density, providing insights for mix design optimization [98].

Apart from these, various mathematical models are used to predict rheological properties such as yield stress and viscosity, which are essential for understanding the behavior of complex fluids like fresh concrete and suspensions. The Bingham model describes a linear relationship between shear stress and shear rate, while the Herschel–Bulkley (H-B) model is a generalized form that incorporates non-Newtonian behavior. Other models include the power law (1929) [99], Eyring (1936) [100], Sisko (1958) [101], Casson (1959) [102], and Williamson (1929) [103] models, among others [104]. Additionally, the Yield Stress Model (YODEL) accounts for microstructural properties such as solid volume fractions, particle size and distribution, maximum packing, percolation threshold, and interparticle forces. It accurately predicts the dependence of yield stress on volume fraction and the inverse relationship between yield stress and particle diameter in particulate suspensions [105,106]. Another widely used modelling technique is the Krieger–Dougherty (K-D) model, which derives dynamic viscosity based on the volume fraction of cement particles, a value that can be determined from the w/c ratio [107,108]. Rajagopalan et al. (2022) [109] investigated the rheological properties of non-blended cementitious suspensions containing PC, fly ash, BFS, and SF. Yield stress was predicted using YODEL, which considers particle-size distribution, interparticle forces, and microstructural parameters. Its predictions closely matched experimental results, with an R^2^ above 0.96. Plastic viscosity was estimated using the K-D equation, which incorporates the maximum packing fraction and intrinsic viscosity, achieving an R^2^ above 0.94. Both models provided accurate, parameter-free predictions, demonstrating their applicability to multimodal powder suspensions.

These studies demonstrated the effectiveness of modelling techniques in predicting the properties of fresh concrete. However, the accuracy of these models can be affected by variations in mix parameters and material properties, highlighting the need for consistent input data and further research to refine and optimize these prediction models for diverse concrete mixtures.

### 3.2. Internal Relative Humidity (IRH) and Degree of Hydration (DOH)

IRH and DOH play crucial roles in determining the properties and performance of concrete, particularly in relation to its early-age behavior and shrinkage. Monitoring IRH during concrete curing helps in understanding the internal stresses and hydration progress, which significantly affects the rate and extent of cement paste hydration [109,110,111,112]. Several researchers have proposed prediction models for IRH and DOH, focusing on various factors, such as internal curing (IC) water content, temperature, w/c ratio, and age of the concrete.

Shen et al. (2015) [113] proposed a formula to calculate IRH in internally cured concrete, considering the IC water content under both sealed and unsealed conditions. The formula for RH as a function of age and IC water content is expressed as:

(2) $$RH\left(t,Q\right)=K_{t}\sqrt{Q}+RH(t,0)$$

where *RH*(*t*,*Q*) is relative humidity; *Q* is IC water content; $K_{t}$ is a parameter related to IRH and IC water content; *t* is concrete age; and *RH*(*t*,0) is the relative humidity of concrete without IC at different ages.

The relationship between IRH and concrete age under unsealed conditions without IC water is modelled as:

(3) $$RH\left(t,0\right)=100-\gamma \times t^{\theta }$$

where *γ* and *θ* are parameters that can be determined with regression analysis.

Under the effects of drying and self-desiccation in unsealed conditions, the relationship between parameter $K_{t}$ and concrete age (*t*) is expressed as follows:

(4) $$K_{t}=\alpha \times (1-e^{-\beta t})$$

where *α* and *β* are parameters that can be determined with regressive analysis.

By adding Equations (3) and (4) into Equation (2), the following equation is derived for relationship between *RH* and age,

(5) $$RH\left(t,Q\right)=\alpha \times (1-e^{-\beta t})\sqrt{Q}+(100-\gamma \times t^{\theta })$$

The accuracy of the experimental results enables the prediction of IRH in internally cured concrete at different ages, considering the IC water content.

Shen et al. (2017) [114] also investigated the effect of pre-wetted lightweight aggregates (LWAs) in mitigating shrinkage in HPC. They developed equations for predicting critical time and working w/c ratios, showing good alignment between the model’s predictions and experimental data. Additionally, the IRH evolution in concrete is divided into two stages: an initial stage with 100% RH (Stage I) and a subsequent decrease in RH (Stage II). The duration of Stage I is defined as the critical time. Prediction equations for IRH under varying w/c ratios and SAP contents showed higher R^2^ values ranging from 0.795 to 0.865, confirming the accuracy of these models in predicting the IRH behavior and early-age shrinkage of internally cured concrete. Similarly to the modelling of concrete with LWA, Shen et al. (2017) [115] proposed several prediction equations related to early-age concrete internally cured with SAPs. They developed a model for the working w/c ratio, considering age as well as effective and IC w/c ratios. Additionally, a prediction model for the critical time of IRH was formulated based on the effective and IC w/c ratios. Furthermore, they proposed a model for IRH in early-age concrete, incorporating the working w/c ratio, critical time, and age. For experimental study, the HPC mixtures were prepared with varied w/c ratios (0.33, 0.40, and 0.50) and SAP dosages (0.05%, 0.15%, and 0.25%). The prediction models were derived using experimental data and demonstrated good accuracy.

In terms of DOH, development of time-dependent models to predict the hydration degree of cement and slag, free water content, specific heat, and total heat generation in slag-incorporated concrete. These models are then integrated to estimate the adiabatic temperature rise in mass concrete containing slag. The hydration degree of cement was determined based on the reactions of C_3_A, C_3_S, C_2_S, and C_4_AF in concrete. A model for slag hydration degree is developed through back-analysis of temperature profiles from slag-containing concrete, combined with findings from selective dissolution tests on slag-cement pastes. This model considers factors such as age, w/b ratio, concrete temperature, slag replacement level, and slag fineness. The model also accounts for the physical acceleration of cement hydration by slag particles by incorporating dispersion effects for C_3_S and C_3_A. Using experimental data, models were developed to calculate free water content and specific heat in slag-cement pastes. The total heat generation model was derived by summing the heat contributions from all reactive compounds in concrete. All models were integrated to simulate adiabatic temperature rise in mass concrete. The model was validated by comparing predicted and experimental results for adiabatic temperature rise in various slag concrete mixtures, demonstrating reasonable accuracy [116].

Furthermore, a mathematical model based on a single kinetic equation was developed to predict the extent of hydration and the microstructural evolution of cement particles. The model accounts for the formation and breakdown of the initial impermeable layer, the activation of chemical reactions, and subsequent diffusion-controlled hydration. Cement particles were assumed to be spherical, with an initial surface layer of hydration products through which external water diffuses, reacts with fresh cement, and produces new hydration products outward. The hydration rate is governed by mass transfer control, where water diffusion plays a key role. Further, an ANN model was employed to evaluate the kinetic factors B, C, D, and kr in the mathematical model for hydration, with input parameters such as cement mineral composition (C_3_S, C_2_S, C_3_A, C_4_AF), average particle radius, cement density, and w/c ratio. The neural network was developed as a multi-input, single-output BPNN with four intermediate layers and a sigmoid activation function. When applied to ordinary Portland cement (OPC) and belite-rich Portland cement (BPC), the model slightly underestimated hydration degrees at early ages compared to experimental data [117]. The numerical reaction-kinetics models, such as phase boundary nucleation-and-growth models, rely on a combination of theoretically derived kinetic mechanisms and assumptions. However, these models fail to provide a priori predictions of cement hydration kinetics, particularly in multicomponent systems where complex chemical interactions between cement, water, and mineral additives occur simultaneously [118].

To address these limitations, an ML-based model was developed to enable rapid and high-fidelity predictions of time-dependent hydration kinetics in both plain and multicomponent cement systems (e.g., binary and ternary systems). This approach utilizes the physicochemical characteristics of the system as input variables. An RF model was rigorously trained on a database of 235 unique hydration kinetics profiles, covering seven synthetic cements and three mineral additives with diverse properties. A modified RF model was then trained on a high-volume dataset containing hydration profiles of hundreds of systems with varying cement compositions and mineral additives (e.g., limestone with different particle size distributions and MK), replacing 0–60% of cement mass. Results indicate that the RF model—once trained with physicochemical inputs—can accurately predict continuous, time-dependent hydration kinetics, outperforming semi-empirical kinetic models that fail to make a priori predictions in complex systems. Furthermore, when combined with Bayesian optimization, the model enables the prediction of optimal cementitious mixtures that satisfy performance (e.g., setting time, 28-day CS) and sustainability (e.g., cement content < 50%) criteria—without requiring a full understanding of underlying kinetic mechanisms [118].

Future expansions of the database, including mortars and concrete with various cementitious materials and aggregates, could further enhance the model’s ability to recommend optimal additives and proportions for achieving the desired DOH. Additionally, incorporating various types of ML models, alongside traditional modelling techniques will not only improve accuracy but also offer more robust predictions.

### 3.3. Porosity

Concrete porosity plays a critical role in determining its durability and mechanical properties. Several methods and models are available to predict and evaluate porosity, some of which are discussed further. Understanding and accurately assessing porosity is essential for designing long-lasting concrete mixes and analyzing the material’s performance across different environmental conditions.

Cao (2023) [88] utilized ML to predict the porosity of HPC comprising SCMs. The dataset consists of 240 records covering 74 unique concrete mix designs, with input parameters including the w/b ratio, binder content, fly ash (%), BFS (%), SP (%), CA/FA ratio, and curing days. Both GBT and RF models were trained, with hyperparameters optimized using Bayesian optimization by minimizing either k-fold cross-validation error or out-of-bag error. Specifically, RF were optimized based on out-of-bag error, while GBT used k-fold cross-validation error. The results indicated that GBT outperformed RF, with XGB achieving the highest accuracy due to its regularization capability, which mitigates overfitting. Sensitivity analysis further highlighted the long-term benefits of fly ash and BFS in reducing concrete porosity. Compared to traditional statistical regression or classical chemo-mechanical hydration models, the proposed ensemble learning approach handled complex concrete compositions and achieved high predictive accuracy (R^2^ = 0.9770, MAPE = 2.97%, and RMSE = 0.431). Predictor importance analysis identified curing duration, w/b ratio, and aggregate content as the most influential factors affecting concrete porosity. Similarly, Le et al. (2022) [119] predicted the effective porosity (EP) of pervious concrete using three distinct ML models—ANN, XGB, and SVM—representing neural networks, tree-based models, and kernel-based models, respectively. Additionally, RFR was included for comparison. The PSO algorithm was applied to tune hyperparameters for all models except ANN, due to its higher computational demand. Instead, a sensitivity analysis was conducted for ANN. Results showed that XGB significantly outperformed the other models, achieving an R^2^ of 0.88 in testing. Furthermore, to account for the role of compaction, the original database was refined to a subset of 36 samples that considered compaction energy. In this case, RFR outperformed XGB, improving accuracy to R^2^ = 0.97.

Further, using six ML algorithms: linear regression, ANN, boosted decision tree regression, RFR, KNN, and SVR, predicted the porosity and permeability of pervious concrete based on mix parameters (compaction energy, aggregate-to-cement ratio, and aggregate size) and ultrasonic pulse velocity (UPV). By utilizing non-destructive measurements and mix design variables, these models enable practical application in the construction industry without requiring theoretical expertise. Results indicated that ANN was the most effective model for predicting porosity (R^2^ = 0.9502 for training and R^2^ = 0.8958 for testing), while boosted decision trees performed best for permeability (R^2^ = 0.9323 for training and R^2^ = 0.7574 for testing). Sensitivity analysis using RFR highlights ultrasonic pulse velocity as the most influential factor in predicting porosity [120].

Zavrtanik et al. (2016) [121] analyzed a database of 17,296 asphalt mixture samples to estimate the air void content in aggregate mixtures of various stone fractions across seven asphalt concrete types produced under EN 13108-1 [122]. The research modeled the relationship between different parameters and air void content using both ANN and MLR. Feedforward neural networks with error back-propagation were employed, utilizing NTR2003 and the WEKA toolkit. Before modelling, outliers were identified, followed by ANN and regression analysis for both individual and combined mixtures. Results showed that linear models performed better for specific asphalt mixtures, while ANNs were more effective in detecting hidden patterns across all mixtures. Overall, feedforward neural networks proved to be a reliable tool for preliminary air void content estimation in aggregate mixtures.

### 3.4. Compressive Strength (CS)

CS is one of the most critical parameters used to assess concrete’s ability to withstand heavy loads. It serves as a key indicator of concrete quality and performance, influencing its suitability for various structural applications. Accurate prediction of CS is essential for optimizing mix designs, ensuring structural integrity, and reducing material costs. To achieve reliable predictions, numerous researchers have explored both numerical methods and ML based approaches. Several of these approaches are discussed in the following sections.

There are various numerical formula-based models used by researchers to predict CS. For example, Abrams’ law relates the CS of workable concrete to the w/c ratio, cement content, air content, and the cement hardening rate [85,123]. In Slater’s model, the strength of concrete changes linearly with the w/c ratio [85]. Bolomey’s formula incorporates cement, water, and the characteristics of aggregates [124], whereas Powers’ gel–space ratio model accounts for the contribution of various hydration products [54,72,125]. Feret’s model considers the absolute volumes of cement, water, occluded air, and the composition of cement [54,72,124].

De Brito et al. (2018) [85] evaluated three original models (Abrams, Slater, and ACI) and two modified models (Bolomey and Feret) for estimating the 28-day CS of concrete made with OPC and NA. However, these models failed to accurately predict CS when aggregate properties were not considered. In all original models, the relationship between actual and calculated strength was weak when the w/c ratio—whether or not accounting for aggregate volume—was the only influencing factor. Significant variations were observed between calculated and experimental strengths, even for concrete made solely with NAs. These discrepancies arose based on the geological type (e.g., limestone, basalt, granite, sandstone) and quality (density, water absorption, abrasion resistance) of the aggregates. Classifying mixes by geological nature or quality reduced these discrepancies. The modified models (Bolomey and Feret) outperformed the original models (Abrams, Slater, and ACI) by incorporating cement strength class as an additional factor. However, Bolomey’s model overlooked the aggregate-to-cement paste volume ratio, while Feret’s model ignored the method of aggregate production (e.g., rolled or crushed). The modified models predicted higher strengths for basalt-containing mixes, followed by those with limestone, quartz, and granite. Additionally, strength calculations tended to be overestimated for lower-quality aggregates. The impact of aggregate properties became even more pronounced when recycled aggregates (RA) were used, due to their heterogeneous nature.

Similarly, Olawuyi (2016) [126] modelled the impact of SAP on concrete strength by combining Bolomey’s formula with Powers’ model. The results showed that the net effect of SAP (positive or negative) depends on the mix design and curing age. SAP addition reduced the CS of HPC, but adjusting for void volume (SAP and air) using Bolomey’s formula and Powers’ gel–space ratio improved the fit within Powers’ model, enabling better prediction of SAP’s effect on CS of HPC.

Meera and Gupta (2020) [127] developed a CS prediction model for foam concrete, incorporating an effective w/c ratio that accounts for foam volume. Their model builds on Abram’s strength-to-water-cement law and introduces an equivalent w/c ratio using an efficiency factor for fly ash, improving the prediction of foam concrete strength. By considering foam effectiveness, the model improves prediction accuracy. When tested on foam concrete mixes from previous studies, it showed higher efficiency and ease of use compared to other models, requiring only data on water, foam, and binder.

Gao et al. (1997) [71] used a Box–Behnken statistical design model to study the impact of SAP/clay composite (SAPC)/cement ratio, SAPC particle size, and cement/water ratio on the mechanical properties of aluminate cement paste and SAPC-modified concrete. For aluminate cement paste, CS increased parabolically with water content, linearly with SAPC particle size, and decreased parabolically with SAPC content. In concrete, compressive strength showed a linear increase with larger SAPC particle size and higher cement/water ratio, while it decreased parabolically with higher SAPC content. The derived equations offered a useful tool for optimizing mix designs and understanding SAPC’s effects on concrete strength and elasticity. Further, Shen et al. (2020) [128] established a prediction model for mechanical properties with increasing age to study the early-age behavior of internally cured HSC with varying SAP contents (0.57–1.14%). The derived equation for predicting time-dependent compressive strength $f_{c}(t)$ is as follows,

(6) $$f_{c}(t)=f_{c,28}\;exp\left\{-\lambda_{1}[{\ln\left(1+(t-t_{0})\right)]}^{-k_{1}}\right\}$$

where $f_{c,28}$ is the 28-day CS, *t* is the age of concrete after pouring, $t_{0}$ is the initial setting time, and $\lambda_{1}$ and $k_{1}$ are the fitting parameters for different concrete mixtures. The predicted values closely aligned with the experimental data.

Chidiac et al. (2013) [129] proposed a comprehensive model for predicting the CS of concrete based on factors such as cement type, DOH, aggregates type and gradation, mixtures proportions, and air content. The CS is predicted using four sub-models: the average paste thickness model, which considers the proportions of the concrete mixture and its packing density; the cement hydration model by Schindler and Folliard (2005) [130], which accounts for the chemical composition and properties of cementitious materials; the paste-to-aggregate bond strength and cement standard strength were modelled following de Larrard’s approach (1999) [131]; and capillary and air pores were modelled according to Popovics (1985) [132], which is a modification of Abrams’ model. The CS model demonstrated a strong fit with experimental data, showing no outliers or patterns, with a standard error of less than 2 MPa for both 3-day and 28-day predictions, and an R^2^ above 0.95. Additionally, while the de Larrard model was the most comprehensive compared to others, its section on cement composition and chemical reactions lacks completeness, which limits its broader applicability.

Zhang et al. (2022) [133] used the relationship between CS and the pore structure of cement-based materials, proposed by Odler and Rößler (1985) [134], to predict CS with a w/c between 0.22 and 0.52.

(7) $$\sigma_{c}=100-k_{1}\;V_{pe}-k_{2}\;V_{pb}-k_{3}V_{ph}$$

where $\sigma_{c}$ is the CS of cement mixed with water absorbing polymer (WAP), $V_{pe}$ is the pore content of the internal curing region in per unit volume cement stone, $V_{pb}$ is the pore volume of the matrix in per unit volume cement stone, $V_{ph}$ is the volume of pores introduced by WAP in the unit volume cement stone, and $k_{1}$, $k_{2}$, and $k_{3}$ are the influence coefficients. The CS was predicted based on the internal-curing width and compared with experimental results, showing only a 7% error. The impact of internal-curing width, size, and mass swelling ratio of WAPs on CS ranged between 80 MPa and 90 MPa, while the dosage and volume swelling rate had a broader influence, ranging from 20 MPa to 90 MPa. Overall, the CS was primarily affected by the dosage and volume swelling rate of WAPs, with higher values leading to lower CS. However, these models have limitations, such as the calibration and influence coefficients, which depend entirely on the quality and quantity of the experimental data [129,133,135].

A regression model was developed using the a/c ratio, w/c ratio, and compaction energy level as input variables. The model demonstrated high accuracy, with an R^2^ of 0.905. However, it did not account for factors such as grading and properties like size, shape, and origin in the CS prediction [136]. Additionally, three models—two linear and one non-linear models—were developed using particle density, RA, resistance to fragmentation—Los Angeles (LA), and water absorption (WA) as independent variables. The linear models achieved R^2^ values of 0.65 and 0.97, while the non-linear model showed an R^2^ of 0.91, indicating a higher predictive capability for the linear model. However, determining the values for certain parameters, such as LA and WA, proved challenging due to the lack of available data in some cases [137]. Furthermore, linear, and logarithmic equations were derived considering rubber content and the w/c ratio. A linear best-fit model accurately predicted density reduction based on rubber content, while logarithmic best-fit curves estimated CS within the tested range (5–50% rubber content, 0.45–0.55 w/c ratio). To further enhance prediction accuracy, factors such as the cement-to-aggregate ratio, initial strength of control samples, and the type and size of recycled rubber aggregates should be considered [138].

Furthermore, in the context of using ML to predict CS, Mohammed et al. (2021) [53] applied various statistical and ML techniques, including LR and NLR, Multi-Logistic Regression, M5P-tree, and ANN (a multi-layer feedforward neural network with a hyperbolic tangent transfer function). Key influencing factors included the fly ash incorporation ratio, fly ash content, w/b ratio, gravel, sand, cement content, and curing ages. Among these models, the M5P-tree demonstrated superior performance, with R values of 0.972 (training) and 0.823 (testing). The prediction errors for Multi-Logistic Regression, LR, M5P-tree, and ANN ranged between 0.03–43%, 0.03–54%, 0.04–33%, and 0.03–41%, respectively. Sensitivity analysis identified curing time as the most critical factor influencing the CS. Similarly, ANN model outperformed all other models (LR, NLR, MLR, and Multi-Logistic Regression), demonstrating the highest accuracy in predicting CS [8,9,81,97,139,140]. In one study, a single ANN was divided into five specialized ANNs based on the relationship between curing temperature, humidity, and concrete strength. ANN-I predicted early strength within 24 h using pouring day conditions. ANN-II and ANN-III estimated strength increments on the second and third days, respectively, using the conditions of the previous days. ANN-IV and ANN-V predicted strength increments on the 7th and 28th days, utilizing temperature and humidity history from days 3–6 and 7–28, respectively. Results confirmed that the ANN effectively predicted CS development, achieving R^2^ values ranging from 0.91 to 0.97 [141].

**Figure 6:**
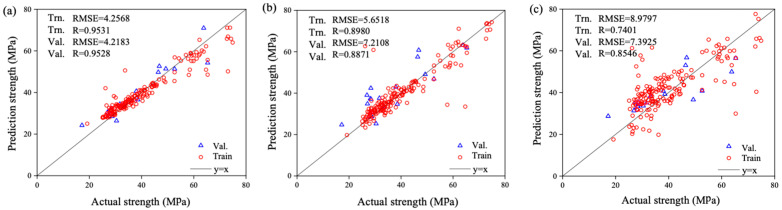
Predicted versus actual UCS values on training and validation sets using ML model [7]. (**a**) BPNN, (**b**) SVM, (**c**) RF.

The CS was also predicted using two NN models: feedforward backpropagation (BP) and cascade correlation (CC), based on eight variables, including sand, w/c ratio, lightweight FA and CA, SF (in solution and as cement addition), SP, and curing period. Results showed that the CC model (architecture 8-6-4) provided slightly more accurate predictions with an R value of 0.982 and learned faster than the BP model [57]. On the other hand, Chen et al. (2024) [7] applied three ML approaches—BPNN, SVM, and RF. To enhance performance, the PSO algorithm was integrated with cross-validation for hyperparameter tuning. After training and modelling, a comparative analysis was conducted to determine the most effective model for optimizing the objective function. Results indicated that BPNN demonstrated the highest predictive accuracy for CS, achieving an R^2^ value of 0.953, a low RMSE of 4.218, and a MAE of 3.419 during validation, as shown in Figure 6, ensuring precise and reliable predictions.

Zhang and Aslani (2021) [142] proposed two predictive models—the regression model and the genetic algorithm (GA)-BPNN Model—for estimating the CS of LWA Concrete (LWAC) with UPV and incorporating parameters such as w/b ratio, maximum aggregate size, maximum LWA size, volumetric ratio of CA/b, volumetric s/a ratio, and replacement ratio. The regression model provided reasonable approximate predictions across different subgroups, with R^2^ values ranging from 0.736 to 0.988. Meanwhile, the GA-BPNN achieved higher accuracy, with R^2^ = 0.958 and RMSE = 4.51. Although GA did not significantly increase the maximum correlation coefficient, however, it notably improved the mean and maximum RMSE, enhancing model stability. A deep convolutional neural network was trained on 380 concrete mix samples and validated against SVM, ANN, and AdaBoost models using an experimentally prepared dataset. The proposed model achieved high accuracy, with R^2^ of 0.973 (training) and 0.967 (testing), demonstrating strong predictive performance and generalization ability. Additionally, sensitivity analysis suggested increasing the w/b ratio reduces CS, while an optimal sand ratio maximizes it, varying with the paste-aggregate ratio [143]. Further development of an optimized deep neural network (DNN) model with five hidden layers and a learning rate of 0.001, achieved a MSE of 28.76 and an R^2^ of 0.89, outperforming traditional regression models [5].

Additionally, ANN (feedforward NN), GEP, and GBT were employed to predict CS. GEP is a soft computing technique that combines genetic algorithms and genetic programming to develop empirical models for estimating concrete properties. It encodes data into linear chromosomes, forming expression trees, with the best-performing chromosomes passed to successive generations until optimal fitness is achieved. The models incorporated easily measurable variables such as concrete density, w/c ratio, and s/c ratio. Among the three models, GBT demonstrated the highest accuracy (R = 0.977, MAE = 1.817, RMSE = 2.69), outperforming ANN and GEP. The study concludes that the GBT model is the most reliable for strength prediction, while the GEP model provides a useful predictive equation [54].

**Figure 7:**
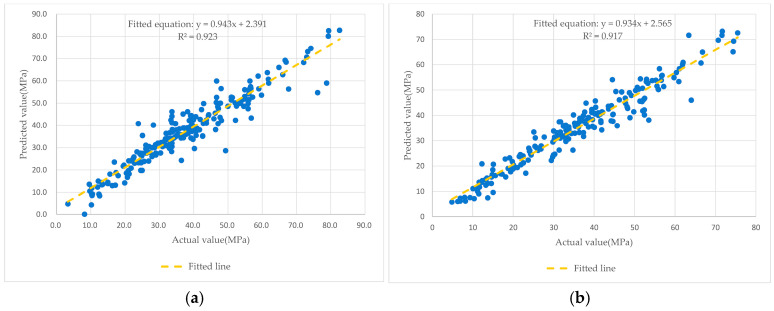
Relation between experimental and predicted CS [68]. (**a**) Validation, (**b**) Testing.

The Gradient Boosting Regression Tree (GBRT) algorithm was applied, considering eight input variables: C, BFS, fly ash, W, SP, CA, FA, and age. The dataset was divided into three parts: 60% for training, 20% for validation, and 20% for testing. The training set was used to develop the prediction model, while the validation set helped fine-tune hyperparameters and prevent overfitting. Finally, the testing set was used to evaluate the model’s performance, ensuring its accuracy and reliability. The GBRT model demonstrated high prediction accuracy in forecasting the CS of concrete, achieving an R^2^ of 0.923 (validation) and 0.917 (testing) (see Figure 7), compared to other modelling techniques (ANN, SVM, RF and AdaBoost). Further validation using a five-fold cross-validation approach confirmed the model’s accuracy and reliability, with R^2^ and RMSE values consistently demonstrating strong performance across all folds [68].

Similarly, Kang et al. (2021) [144] found that boosting- and tree-based algorithms performed best, while KNN, linear, ridge, lasso regressor, SVR, and MLP models performed worst. The study also identified the w/c ratio and SF content as the most significant factors influencing CS. Rathakrishnan et al. (2022) [77] evaluated five boosting ML (BML) algorithms—LightGBM, CatBoost, GBR, AdaBoost, and XGB—using Random Search optimization and default hyperparameter models for comparison. The dataset included seven concrete components (FA, CA, OPC, GGBS, SF, W, SP, and MC) with CS measured at 7, 28, 56, and 91 days. Among the algorithms, GBR achieved the highest accuracy (R^2^ = 0.96) and the lowest error (MAE = 2.73, RMSE = 3.40), making it the best-performing model for predicting concrete CS.

Four ML models, DT, RF, XGB, and Explainable Boosting Machine (EBM) were used along with Bayesian optimization technique to efficiently select algorithms and tune hyperparameters, thereby reducing model-building time. A dataset of 1030 test samples was used, with input parameters including OPC, fly ash, BFS, W, SP, CA, FA, and concrete curing age. Among the four ML models, EBM achieved the best predictive performance (R^2^ = 0.93, RMSE = 4.33, MAE = 3.10). EBM also exhibited the smallest interquartile range (IQR), indicating lower dispersion in predictions, while the DT had the largest dispersion. Additionally, the average predictions of EBM and XGB were closest to the ideal value of 1.0 [145].

On the other hand, Ullah et al. (2022) [146] compared SVM as an individual learner with ensemble learners, including bagging, boosting, and RF, using a dataset of 191 samples. The key input variables were C, sand content, w/c ratio, and foam volume. Among these models, RF outperformed the others, achieving R^2^ = 0.96 with the lowest errors (MAE = 1.84 MPa, RMSE = 2.52 MPa). An evolved SVR (ESVR) model, tuned by beetle antennae search (BAS)—a meta-heuristic optimization algorithm inspired by beetles’ behavior—was used to predict the 28D UCS of pervious concrete. The input variables were w/c ratio, a/c ratio, and aggregate size. The BAS outperformed random hyperparameter selection in reliability and efficiency, with the ESVR model showing higher predictive capability than baseline models. However, this study was limited by a relatively small dataset and variables selection [86].

Tao (2024) [12] developed predictive models including BPNN, RF, XGB, and HEStack method to estimate the uniaxial CS of nano-enhanced concrete. A dataset of 94 samples, incorporating eight input parameters—w/c ratio, carbon nanotubes, NS, nano-clay, nano-aluminum, C, CA, and FA—was used for training and evaluation. HEStack achieved the highest R^2^ value of 0.9924, with a low MAPE of 2.84%, RMSE of 1.6495, RSR of 0.0874, and NMBE of 0.0064—outperforming BPNN (R^2^ = 0.9356), RF (R^2^ = 0.9706), and XGB (R^2^ = 0.9884). These results established HEStack as the most reliable model for predicting nano-modified concrete properties.

Hybrid modelling techniques have emerged as powerful tools for improving predictive accuracy by combining multiple algorithms to optimize performance. Pham et al. (2015) [84] predicted the CS of HPC using a dataset of 293 HPC mixes with a hybrid model that combined the FFA and LS-SVR. LS-SVR establishes the functional relationship between HPC components and CS, while FFA optimizes LS-SVR for improved accuracy and generalization. The hybrid model effectively predicted strength with R^2^ values of 0.92 (training) and 0.89 (testing). Similarly, Yaseen et al. (2018) [147] proposed several models—Extreme Learning Machine (ELM), Multivariate Adaptive Regression Spline (MARS), M5 Tree, and SVR models—to predict the CS of lightweight foamed concrete, using input parameters such as C, oven-dry density, w/b ratio, and foamed volume. The predictive accuracy of the models was evaluated using statistical indicators, showing that ELM (R^2^ = 0.983) outperformed MARS (R^2^ = 0.956), M5 Tree (R^2^ = 0.982), and SVR (R^2^ = 0.946).

Shishegaran et al. (2021) [148] developed a hybrid model using UPV and Rebound Number (RN) data. A dataset of 516 points from eight studies was utilized. The High Correlated Variables Creator Machine (HCVCM) was employed to generate new variables with stronger correlations to the output, thereby improving the prediction accuracy. The study incorporated three individual models—Step-by-Step Regression (SBSR), GEP, and ANFIS—alongside three hybrid models: HCVCM-SBSR, HCVCM-GEP, and HCVCM-ANFIS. SBSR is a regression model designed to enhance accuracy by optimizing the coefficient of determination between input and output. Results indicated that HCVCM-ANFIS outperformed all other models, achieving improvements of 5% in R^2^, 10% in RMSE, 3% in NMSE, 20% in MAPE, and 7% in the maximum negative error, demonstrating superior predictive accuracy. Kashem and Das (2023) [149] employed supervised hybrid ML models, including XGBR-BR, SVR-RFR, and GBR-DTR, and used SHAP to analyze input variables and explain predictions. The XGBR-BR model achieved high prediction efficiency, with 99% accuracy in training and 96% in testing, while all models showed error margins within ±10%. SHAP dependence plots for XGBR-BR revealed that C, W, and SP were the most influential factors for HSC, with C having the highest mean SHAP value (5.79) and FA and CA showing lower impact on CS.

The RF algorithm, known for handling complex systems and multiple parameters, was integrated with optimization techniques like the Rider Optimization Algorithm (ROA), Black Widow Optimization Algorithm (BWOA), and COOT Optimization Algorithm (COA) to enhance accuracy. Results showed that the RF model optimized with COA (RFCO) outperformed the others, achieving the lowest error rates and the best RMSE values in both training and testing, demonstrating reliable forecasting capabilities. Despite consistent errors across all models, the study suggests that RF hybrid models, especially RFCO, provides highly effective and accurate predictions for engineering applications [150]. The authors of [151] developed a novel RF–grey wolf optimization (GWO)–XGB ensemble ML model for landscape geopolymer concrete (GePoCo). Using a dataset of 156 samples and 15 variables, the model combined RF, GWO, and XGB for improved prediction. The ensemble approach involved stacking multiple RF models with different hyperparameters, generating a new dataset, and then training an XGB model (RF–XGB). The GWO algorithm was then applied to optimize the RF–XGB model, leading to the RF–GWO–XGB model, which was compared against stand-alone RF, XGB, and a GWO–XGB hybrid model. The RF–GWO–XGB model significantly outperformed standalone RF, XGB, and GWO–XGB models, achieving RMSE values of 1.712 and 3.485 and R^2^ values of 0.983 and 0.981, demonstrating superior accuracy.

A few modelling studies have explored the relationship between porosity and CS. These studies aiming to understand how porosity influences the strength. Numerous studies have also focused on developing strength–porosity equations for porous materials using various mathematical models.

(8) $$\sigma_{c}=\sigma_{c,0}{\left(1-P\right)}^{n}\;\;\;\mathrm{Balshin}$$

(9) $$\sigma_{c}=\sigma_{c,0}e^{-k_{r}P}\;\;\;\mathrm{Ryshkewitch}$$

(10) $$\sigma_{c}=K_{s}\;In(P*/P)\;\;\;\mathrm{Schiller}$$

(11) $$\sigma_{c}=\sigma_{c,0}-K_{H}\;P\;\;\;\mathrm{Hasselmann}$$

where *P* is the total porosity, $\sigma_{c}$ is the CS of foamed concrete, $\sigma_{c,0}$ is the CS at zero porosity, and P∗, $K_{s}$, $k_{r}$, *n*, $K_{H}$ are the empirical constants derived from regression analysis of laboratory data [21]. Kearsley and Wainwright (2002) [73] studied the effects of replacing up to 75% of cement by weight with both classified and unclassified fly ash in foamed concrete. The research focused on the relationship between porosity and compressive strength, developing mathematical models (including those by Balshin, Ryshkewitch, Schiller, and Hasselmann) to describe this correlation. The findings indicated that the CS of foamed concrete depends on both porosity and age. Among various models, the multiplicative equation derived by Balshin provided the best fit across all ages up to one year. Additionally, Hoff’s equation was found effective for predicting the CS of foamed concrete mixtures with high fly ash content. Since CS is influenced by the total volume of voids—such as entrapped air, capillary pores, gel pores, and entrained air—several models have been proposed to describe the strength–porosity relationship.

Further, the relationship between effective CS and porosity was derived using a simplified center pore model. To account for pore structure influence, pore size was selected as a key parameter, and an influence function was proposed based on correlation analysis. The total influence coefficient was determined by combining the pore size distribution and influence function. Incorporating this coefficient into strength–porosity relations led to explicit formulae for predicting effective strengths. Comparisons with analytical and experimental data confirmed the model’s reliability, showing that porosity has a greater impact on CS than TS, and that reducing pore size enhances concrete strength at the same porosity level [152]. Existing equations (linear, power, exponential, and logarithmic) relating CS and porosity for cement-based materials were reviewed, with a potential equation for porous concrete was assessed through experimental data fitting [83,153]. A new model based on Griffith’s fracture theory was proposed, showing a much stronger relationship between CS and porosity, with an R^2^ value up to 0.99. This semi-empirical model significantly outperformed the exponential equation, demonstrating strong potential for accurately predicting the CS of porous concrete based on porosity [83].

Each variable in the mix design influences the modeling process, whether through numerical methods or ML approaches. The wide variety of input variables, and decisions regarding their inclusion or exclusion, can significantly affect the accuracy of model predictions. ML techniques are widely adopted by researchers, ranging from non-ensemble and ensemble models to hybrid approaches for predicting CS. While each modeling technique has its limitations, ML methods have generally demonstrated higher accuracy than traditional numerical approaches. Moreover, when predicting the CS of concrete incorporating innovative materials—such as internal curing agents like superabsorbent polymers, lightweight aggregates, and others—it is essential to consider their absorption characteristics to achieve more reliable results.

### 3.5. Tensile Strength (TS)

The safety of concrete structures under impact and impulsive loads largely depends on their TS and stress-strain behavior. These properties influence cracking, the bond between concrete and reinforcing steel, and shear resistance. Accurate prediction of TS is essential for ensuring the durability and stability of such structures [154].

Sim and Yang (2012) [155] assessed the accuracy of various code-based equations (ACI 318-11 (2011) [156], CEB-FIP (1999) [157], EC2 EN 1992-1-1 (2004), NZS 3101 (2006) [158])—as well as researcher-proposed models by Li and Ansari (2000) [159]; Zheng et al. (2001) [160]; Carneiro and Barcellos (1953) [161]; Slate et al. (1986) [162]; Zhang and Gjvorv (1991) [163] for predicting concrete tensile capacities, including direct TS, splitting TS, and modulus of rupture. Using a comprehensive database of 361 lightweight concrete (LWC), 1335 normal-weight concrete (NWC), and 221 heavyweight concrete (HWC) specimens, the study highlights limitations in existing models. Most equations relate TS to CS, primarily based on limited NWC test data. However, findings indicated that concrete unit weight significantly influences tensile capacity. Discrepancies between experimental results and model predictions increase when the unit weight falls below 2100 kg/m^3^ or the CS exceeds 50 MPa. To address these inconsistencies, the authors proposed new models that incorporate unit weight, resulting in more accurate predictions. Zain et al. (2002) [164] derived equations for predicting split TS based on the relationship between CS, w/b ratio, and concrete age, using regression analysis on the experiment data. The experimentally obtained split TS values closely matched the predicted values, with the average ratio of experimental to predicted results approaching unity.

The SVM model was developed, trained, and tested using experimental data from existing literature, incorporating both RBF and polynomial functions during the training process. The predicted results from the SVM model were compared with values from building codes (CEB-FIP (1991) [28], ACI 363R-92 (1992) [165], ACI 318-99 (1999) [166]) and other models, including GEP and regression analysis. These comparisons demonstrated that SVM is a reliable tool for predicting splitting TS from CS, with the highest R^2^ values in testing being 0.8815 for RBF and 0.8823 for polynomial kernel [167]. Additionally, models such as NLR, ANN, SVM, and the M5 model tree were used to predict the TS of concrete, both with and without steel fiber. The results showed that all four models performed well, with ANN (R = 0.943), M5 model tree (R = 0.951), and SVM (R = 0.946), outperforming NLR (R = 0.912). Among previously developed models, those based on ML techniques, such as GEP-III, SVM with radial basis function (RBF), and SVM with polynomial function, showed superior performance compared to NLR-based models [168].

Furthermore, predictions were carried out using five models: DNN1 (10-30-20-1) (10 input variables, 30 neurons in first hidden layer, 20 neurons in second, and one output), DNN2 (10-30-20-10-1), one optimized Gaussian process regression (OGPR), GEP 1 (four gene, 60 chromosomes, and 10 head size), GEP 2 (five gene, 70 chromosomes, 12 head size). DNN2 achieved the highest accuracy (R^2^ = 0.94), outperforming OGPR and GEP2 by 3.3% and 13.5%, respectively, and showing 20.32% and 31.5% better performance in MAE. Similarly, DNN2 and GEP2 outperformed DNN1 and GEP1 by 9.3% and 9.21% in R^2^. Sensitivity analysis identified C content, natural CA, RA density, and SP as the most influential factors affecting tensile strength [169].

Ullah et al. (2025) [170] combined SVR models with optimization techniques such as the FFA, GWO, and PSO to develop hybrid models, comparing them with RF and DT models for forecasting the properties of basalt fiber reinforced concrete (BFRC). Among the models, SVR-PSO performed best, achieving the highest R^2^ value of 0.954, outperforming SVR-FFA (R^2^ = 0.944) and SVR-GWO (R^2^ = 0.930). The RF attained an R^2^ value of 0.918, while the DT model had the lowest R^2^ value of 0.897. SHAP analysis identified fine aggregate (FA) as the most influential factor in predicting TS. PDP revealed that both fine coarse aggregate (FC) and CA positively influence TS. Additionally, a user-friendly graphical interface was developed to facilitate the prediction of CS and TS. Overall, the proposed hybrid models demonstrated strong potential for predicting the durability characteristics of BFRC. Further, the performance of the ANN model was compared to various ML models, including Counter Propagation Neural Nets (CPNN), Radial Basis Function Neural Network (RBFNN), KNN, XGB, CatBoost, DT, RF, Extra Trees (ET), LightGBM, AdaBoost, Bagging, Gaussian Process (GP), SVM. These models used four input features—W, C, sand, and gravel content. The ANN model outperformed all others, yielding more accurate predictions (R^2^ = 0.9397) with lower loss. Sensitivity analysis further revealed that cement content as the most influential factor in predicting concrete’s TS [171].

### 3.6. Flexural Strength (FS)

Flexural strength is a crucial engineering property of concrete, playing a key role in pavement design, crack control, and prestressed concrete applications. Predicting flexural strength is especially valuable for engineering practices that involve fiber reinforced concrete, as well as natural pozzolana and limestone-blended concrete [172].

Lok and Xiao (1999) [173] presented a constitutive model for accurately predicting the flexural response of steel fiber reinforced concrete (SFRC). Explicit formulas were derived for estimate both the first crack strength and the ultimate flexural strength. Key parameters included the direct tensile strength, residual strength, and the product of bond strength ($\tau_{d}$) and fiber aspect ratio (L/d). Both the bond–aspect ratio index $\tau_{d}$ (L/d) and fiber volume fraction significantly influenced SFRC’s flexural behavior. The model’s predictions showed strong agreement with experimental data from SFRC beams and slabs. To further streamline the prediction process, the behavior of parameters—ultimate FS, TS, and $\tau_{d}$ (L/d)—were analyzed over a practical range of fiber volume fractions. This simplified model’s predictions closely matched both the detailed analytical model and experimental results, proving effective for assessing SFRC flexural strength. Similarly, Al-Taan and Wadie (2010) [174] developed a predictive procedure based on the ACI code strain and compression stress block, and also accounting for the enhanced flexural capacity provided by steel fibers in the tension zone. To account this influence key fiber characteristics—including orientation, volume fraction, shape, and dimensions—were considered. The proposed method was evaluated alongside existing methods (Henager and Doherty, Swamy and Al-Taan, and Jindal) using 90 published test results covering a wide range of beam sizes, concrete strengths, reinforcement ratios, fiber properties, and yield strengths. The new approach outperformed the other methods, yielding a calculated-to-experimental strength ratio of 0.99 with a coefficient of variation of 11.2% and exhibited minimal deviation from a normal distribution.

Further, Awodiji and Sule (2021) [175] developed a multivariate regression model to predict the FS of lime-cement concrete beams, water, cement, hydrated lime, river sand, and granite chippings. Utilizing a total of twenty mix ratios—ten for model development and ten for validating. Model adequacy was confirmed at a 95% confidence level using a *t*-test, with a calculated t-value of −1.3342, which fell below the critical value of 2.2622, with an average prediction error of 14.3% compared to experimental results. Additionally, a Visual Basic application was developed using Visual Studio 2015 to aid in selecting mix ratios to achieve a desired FS within the studied range.

In addition to mathematical modelling, ML techniques were also been explored to predict the FS more effectively. Two ML techniques, ANN and GEP, were developed using input parameters: C, limestone, natural pozzolana, and curing ages. The GEP calculated FS using the sum of three expression trees, automatically selecting relevant input variables to generate predictive equations. In contrast, ANN utilized all input parameters by default and lacks automatic variable selection. Both models demonstrated high accuracy, with correlation coefficients of 0.98 (GEP) and 0.99 (ANN), confirming their reliability in evaluating the FS of ternary blended concrete [172]. Similarly, ANN model demonstrated superior performance with R, MAE, and RMSE values of 0.99, 5.67, and 7.37, respectively, compared to the RF regression model, which recorded 0.97, 7.63, and 8.02 for the training data [176].

**Figure 8:**
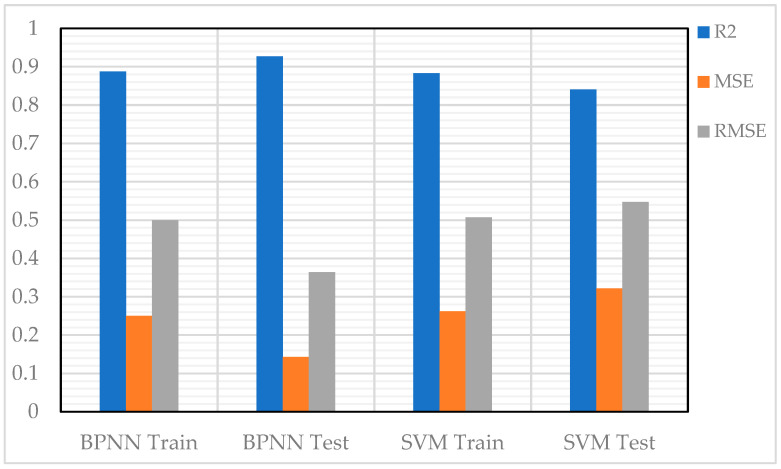
Comparison between actual and predicted FS [76].

Furthermore, BPNN and SVM models were developed using the w/b ratio, fly ash, GGBS, sand ratio, gravel gradation, and aging time as input parameters. During training phase, the R^2^ values were 0.888 for BPNN and 0.883 for SVM, as shown in Figure 8, indicating strong model performance. In testing phase, BPNN demonstrated superior accuracy (MSE = 0.143, R^2^ = 0.927), outperforming SVM (MSE = 0.322, R^2^ = 0.841). The BPNN and SVM predicted values were also compared with findings from the empirical equation in the Chinese Code (2003), further reaffirming that BPNN had the highest accuracy [76]. Conversely, the MLP model exhibited the poorest performance (RMSE = 3.4133, MAE = 2.7871), compared to the GB algorithm, which achieved the best performance (RMSE = 1.5111, MAE = 1.1841). The top five models—GB, XGB, RF, AdaBoost, and DT regressors—produced nearly identical predictive values, closely matching the measured data. Despite some data deviations, their results remained highly reliable. In contrast, linear, ridge, support vector, lasso regressor, and MLP models significantly deviated from actual FS values due to the complexity of the concrete mix and the dataset’s limited size. Feature importance analysis of the top five models confirmed that SF and fiber volume fraction were the most critical variables in predicting FS [144].

Four modelling techniques GEP, ANN, M5P, and RF were used with variables such as C, MK, w/c ratio, FA, CA, SP, and curing days. The results indicated that RF performed the best, achieving an R^2^ value of 0.99, compared to GEP (0.93), ANN (0.97), and M5P (0.87) in the testing phase. The sensitivity analysis ranked the w/b ratio as the most influential parameter, followed by curing days, SP, C, MK, FA, and CA [177].

Supervised ML approaches, including DT-Bagging, DT-GB, DT-AdaBoost, and DT-XGB employed to predict FS of Ultra-High-Performance Concrete (UHSC). The input factors include C, sand, W, SF, fly ash contents, curing time, and the aspect ratio of steel fiber. Among these models, DT-Bagging achieved the highest R^2^ (0.95) and the lowest RMSE and MAE, indicating superior performance compared to DT-AdaBoost (0.93), DT-GB (0.93) and DT-XGB (0.85). Additionally, SHAP analysis revealed that steel fiber content has the most significant positive impact on UHSC FS [69]. Similarly, a GB model demonstrated higher accuracy with an R^2^ of 0.91, outperforming SVM (0.75) and MLP (0.71) in FS predicting [178]. Amin et al. (2023) [179] reported that the SVM model achieved an R^2^ of 0.88, while the BR technique exhibited greater precision in predicting the FS of cementitious composites incorporating waste glass powder.

Finally, XGB, LightGBM, and a hybrid XGB-LightGBM model were employed, with grid search used to optimize hyperparameters. The XGB-LightGBM model achieved R^2^ values of 0.9724 (training) and 0.844 (testing), which was higher than XGB (0.9762/0.819) and LightGBM (0.9519/0.823) for FS prediction. The XGB-LightGBM model achieved MAE values of approximately 0.75 (training) and 2.12 (testing), outperforming XGB (2.25) and LGB (2.36) in the testing phase. The SHAP analysis further highlighted the significant influence of curing age and steel fiber content on UHPC strength [180].

### 3.7. Elastic Modulus (EM)

An ANFIS model and three optimized nonlinear regression models (Nonlin1, Nonlin2, and Nonlin3) were developed to predict the EM of normal strength concrete (NSC) and high strength concrete (HSC) [181]. The nonlinear regression models were optimized using the Differential Evolution (DE) algorithm, which refines solutions through mutation, crossover, and selection. The optimal nonlinear regression model was created by combining the nonlinear regression with equations from building codes (ACI 318-95 (1995) [182] and TS 500 (2000) [183]) as follows:

(12) $$E_{c}=\;a{\;(f_{c}+\;b)}^{c}+d\;\;\;\;\;b\;\mathrm{and}/\mathrm{or}\;d\;=\;0$$

Here, *b* and *d* act as intercepts, with only one of them included at a time in the model. For instance, the ACI formula for NSC can be derived by setting both *b* and *d* to zero ($E_{c}=a{\left(f_{c}\right)}^{c}$), while the ACI formula for HSC is obtained by setting *b* to zero ($E_{c}=\;a{\;(f_{c})}^{c}+d$). In Nonlin1, both *b* and *d* were set to zero, and the DE algorithm optimized *a* and *c* to minimize RMSE, yielding values of 2.12 MPa (training) and 2.33 MPa (testing). In Nonlin2, *d* was set to zero, while in Nonlin3, *b* was set to zero. For HSC, the Nonlin1 model showed RMSE and MAPE values of 2.33 and 0.044 in testing, respectively. Nonlin2 had RMSE and MAPE values of 2.40 and 0.047 in testing, while Nonlin3 displayed RMSE and MAPE values of 2.33 and 0.044, respectively. For NSC, Nonlin1 yielded RMSE and MAPE values of 3.18 and 0.110, respectively. Nonlin2 achieved RMSE and MAPE values of 3.36 and 0.115, while Nonlin3 showed the lowest RMSE and MAPE values of 3.14 and 0.108, respectively. In contrast, ANFIS model demonstrated RMSE and MAPE values of 2.79 and 0.089 for NSC, and 2.20 and 0.041 for HSC during testing. These results confirmed that ANFIS outperformed both nonlinear regression and most literature models, making it a reliable approach for EM prediction.

**Figure 9:**
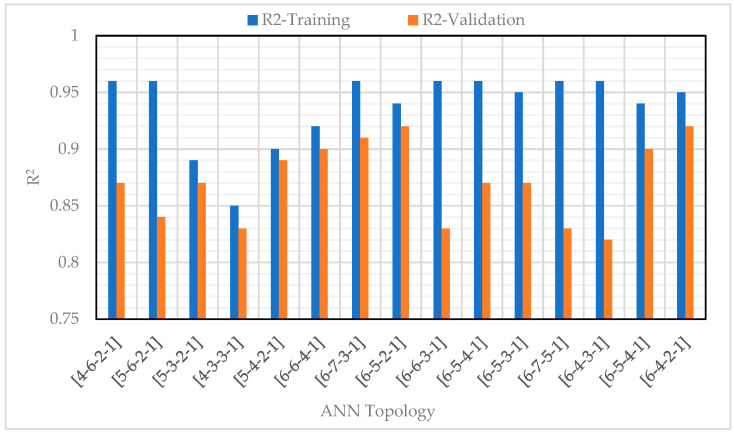
Comparison between various ANN Topologies [184].

Multivariable regression analysis, using linear, polynomial, rational, and exponential functions, was also employed to propose a formulation for predicting the EM. Additionally, ANN model was developed. The predictor variables were C, w/c ratio, replacement of natural aggregate by recycled coarse aggregate ratio (RCA), FA/C ratio, total aggregate/C ratio, and CA/C ratio. A feedforward neural network trained using the Levenberg–Marquardt backpropagation algorithm was evaluated across 15 ANN topologies, with the 6-4-2-1 architecture (input—first hidden layer—second hidden layer—output) performing best. This model achieved R^2^ = 0.95 during training and 0.92 during validation, as shown in Figure 9. In comparison, the mathematical formulation yielded R^2^ = 0.88, with a maximum error of 3.67 GPa [184].

Ahmadi and Kioumarsi (2023) [185] combined ANNs and PSO algorithms to create an innovative hybrid NN-based model for determining the EM of NSC and HSC. Two models, model 1 with one intermediate layer with two neurons, whereas model 2 involved three neurons in the intermediate layer were developed. Both models used concrete compressive strength as a key parameter. The predictions were compared with established code-based formulas, including ACI 318 (2019) [186], ACI 363 (2010) [187], CEB-FIB (1990), and NS 3473 (1998) [188]. Model 1 demonstrated better accuracy in predicting the EM of HSC (MAPE= 4.98%, max error= 25%) compared to NSC (MAPE= 8.33%, max error= 20%). Model 2 outperformed existing relations, with overall MAPE values of 9.37% (NSC) and 5.19% (HSC), and most predictions having errors below 15%. Among existing models, BS 8110 [189] performed best for NSC (MAPE = 10.68%), while CEB-FIB (1990) was most accurate for HSC (MAPE = 5.86%). The results demonstrated that the proposed model performed excellently, offering a reliable method for determining the EM.

Two advanced ML techniques, XGB and AdaBoost regression, were implemented in three modes: individual, hybrid, and ensemble-hybrid. Phasor Particle Swarm Optimization (PPSO) and Chaos Game Optimization (CGO) were applied in hybrid and ensemble-hybrid modes to refine results, minimize errors, and enhance precision. Results showed that PPSO outperformed CGO in optimization and accuracy improvement. Among the models, the XGB-PPSO hybrid demonstrated the best performance, achieving an R^2^ of 0.996 and an RMSE of 0.336, making it an effective ML approach for predicting the EM of recycled aggregate concrete [190].

Zhang et al. (2025) [191] studied three prediction models—BPNN, RBF-NN, and RF model. The hidden layer of each model was optimized through orthogonal testing. The RF feature importance analysis method was used to select the most relevant input parameters, eliminate weakly correlated variables, and reduce the complexity of the prediction model. The results showed that the RBF model outperformed the others, achieving R^2^ of 0.9988, RMSE of 0.04331, MAE of 0.02995, and MSE of 0.01876.

### 3.8. Shrinkage

Concrete experiences volume changes throughout its service life, including shrinkage, which can lead to cracking. There four main types of concrete shrinkage are plastic, autogenous, carbonation, and drying shrinkage (DS). In reinforced concrete, shrinkage-induced cracking can create direct pathways for chloride ions to reach the reinforcing steel, leading to corrosion. This corrosion further contributes to cracking, spalling, and delamination of the concrete [192]. The prediction of shrinkage properties is discussed further using various modelling techniques.

#### 3.8.1. Autogenous Shrinkage (AS)

Several prediction models exist for evaluating AS development in concrete over time. The CEB-FIP considers the CS of concrete and parameters based on the type of cement [157,193]. EN-1992 accounts for both the CS and the composition of cement [193,194]. The Tazawa and Miyazawa [195] model evaluates the influence of cement, chemical admixture, mineral admixtures, and the w/c ratio. Jonasson and Hedlund and Dilger and Wang models consider the w/b ratio [194,196,197]. The model developed by Lee et al. incorporates the w/b ratio, parameters related to BFS, and time at an UPV of 1500 m/s [194,198].

Yoo et al. (2018) [194] simulated AS behavior of high-performance fiber-reinforced cementitious composites (HPFRCC) using several prediction models from the literature. The CEB-FIP, EN-1992, and Jonasson and Hedlund [196] models significantly underestimated AS, while the Tazawa and Miyazawa model overestimated it. In contrast, the Dilger and Wang [197] and Lee et al. [198] models reasonably predicted the ultimate AS strains, although the actual AS progression over time was not well aligned with the predictive values. The shape of the shrinkage development curves closely resembled those of the CEB-FIP and EN-1992 models. Based on these findings, new empirical equations (from (13) to (16)) were proposed through nonlinear regression analysis to improve the accuracy of AS predictions for HPFRCCs.

(13) $${ɛ}_{as}(t)=\gamma {ɛ}_{as\infty }\beta (t)$$

(14) $${ɛ}_{as\infty }=-2300\times exp[-7.2\;(W/B)]\;\mathrm{for}\;\mathrm{SF}$$

(15) $${ɛ}_{as\infty }=-2100\times exp[-7.2\;(W/B)]\;\mathrm{for}\;\mathrm{SF}\;\mathrm{and}\;\mathrm{GGBS}$$

(16) $$\beta (t)=1-exp\;(-0.65\sqrt{t})$$

where γ is a coefficient to describe to the effect of shrinkage reducing admixture (SRA), ${ɛ}_{as\infty }$ is the ultimate autogenous shrinkage, β(t) is the development function of AS. The AS behavior of all HPFRCCs, cured at both ambient and heated temperatures, was accurately simulated using the equivalent age. The steep increase in shrinkage during heat curing was precisely captured. However, these new models are not applicable to other types of concrete, particularly those containing coarse aggregates or different w/b ratios, fiber amounts, and fiber types [194].

Liu et al. (2021) [199] conducted predictions using various models and compared these predictions with experimental results. They found that the models developed by the Federal Highway Administration (FHWA) [200], Dilger and Wang [197], and Yoo et al. [194] were not effective in predicting the AS of internally cured UHPC. While the FHWA and Dilger and Wang models produced similar predicted shrinkage values, the equations proposed by Yoo et al. overestimated shrinkage. The authors introduced a model that utilizes heat of hydration and IRH data, considering two stages. In Stage 1 (IRH = 100%), AS was predicted based on its relationship with hydration heat. In Stage 2 (IRH < 100%), IRH data was used to refine the model, resulting in a straightforward two-stage autogenous shrinkage model, as shown below:

(17) $${ɛ}_{as}=A\times H+B\times \;H^{2},\;\mathrm{when}\;\mathrm{IRH}\;=\;\mathrm{100}\%$$

(18) $${ɛ}_{as}=-k\times RH+b,\;\mathrm{when}\;\mathrm{IRH}\;˂\;\mathrm{100}\%$$

where ${ɛ}_{as}$ is the autogenous shrinkage of cement-based materials, *A*, *B*, *C*, *k*, and *b* are parameters obtained through data fitting, and H is the increase of hydration heat within a certain time.

The predicted 28-day AS results of SAP-modified concrete, based on the CEB-FIP model, were less than 100 × 10^−6^ and developed gradually, similar to the prediction from the EN-1992 model. However, both models underestimated AS compared to the experimental results. Among the existing models, Tazawa’s model exhibited the smallest deviation from the test data. To improve accuracy, a new prediction model was developed by adapting Tazawa’s model and refining using experimental data. The minimal deviations between the predicted and experimental AS results indicated that the new model provides a reliable estimation of AS in internally cured concrete with SAP. This proposed model can estimate the AS for internally cured concrete with varying SAP dosages from the initial setting to 28 days, accounting for early-age expansion. However, its generalizability is limited due to its reliance on specific test data for internally cured and reference concrete, as well as the absence of a universally applicable model for all concrete compositions [193]. Furthermore, Shen et al. (2020) [201] proposed three models to predict early-age AS strain and ultrasonic velocity of internally cured high-strength concrete (ICC) reinforced with Barchip fiber, considering fiber volume percentage and age. Additionally, a model was developed to estimate AS strain based on ultrasonic velocity at different ages, including 28 days without Barchip fiber, while another model incorporated an age-dependent ultrasonic velocity parameter for ICC with Barchip fiber. The prediction results presented good agreement with the experimental results.

Another modelling technique is the B4 model [202,203], which improves upon the B3 model by separating shrinkage into DS and AS components [202]. Židanavičius et al. (2023) [204] evaluated concrete shrinkage predictions using both the EC2 and B4 models [202,205], comparing their results with experimental data. According to EC2, the specimen shrinkage is influenced by several factors, including the concrete’s CS, the type of cement, the RH, and the cross-sectional parameter. In contrast, the B4 model not only considers cylinder CS as the primary parameter influencing concrete shrinkage but also accounts for the concrete mixture composition. The predicted shrinkage values by both models align closely with the experimental results when the RH is between 70% and 80% of the external conditions. Moreover, the B4 model, which offer a more detailed parameter assessment than EC2, was further modified (B4-mod) to account for variable humidity and the effects of quicklime. It was assumed that quicklime’s impact on shrinkage depends on the concrete composition, suggesting that the shrinkage strain component may need adjustment for different mixtures. For a specimen with a 5% quicklime additive, the experimental shrinkage curve closely matched the theoretical predictions from the B4-mod model, with differences of up to 12% after 150 days of curing.

A new numerical approach was developed to predict the AS of alkali-activated concrete (AAC) based on slag and fly ash [206]. This model extends Pickett’s model to enhance AS predictions in AAC. Since existing analytical and numerical models for OPC concrete showed significant discrepancies when applied to AAC, the proposed method addresses these limitations by incorporating the creep behavior of alkali-activated slag (AAS) and alkali-activated slag-fly ash (AASF), along with the restraining effect of aggregates. This model accounts for the pronounced viscoelasticity of alkali-activated materials (AAM) paste and separately analyses the restraining effects of aggregates on both elastic and creep deformations. The extended Pickett model demonstrates strong agreement with experimental data. While designed for AAC, it can be applicable to any concrete where creep significantly influences shrinkage behavior. AS was examined in cement paste and concrete under sealed conditions at room temperature. Further, Wei et al. (2011) [90] analyzed the effects of w/b ratio (0.35, 0.40, 0.45), BFS content (0, 30, 50%), and aggregate content (40%) on shrinkage development over 90 days, beginning 10 h after mixing. Self-desiccation (pore humidity reduction) was predicted using the HYMOSTRUC model. The influence of the w/b ratio on shrinkage development was normalized using shrinkage versus pore humidity curves for Portland cement paste. Additionally, the effect of aggregates on AS followed a Pickett model originally developed for DS. Further, a unified shrinkage model integrating autogenous and DS was proposed, incorporating RH of pore drying, aggregate volume fraction, and the restraint factor in concrete. The restraint factor is also determined based on AS results.

**Figure 10:**
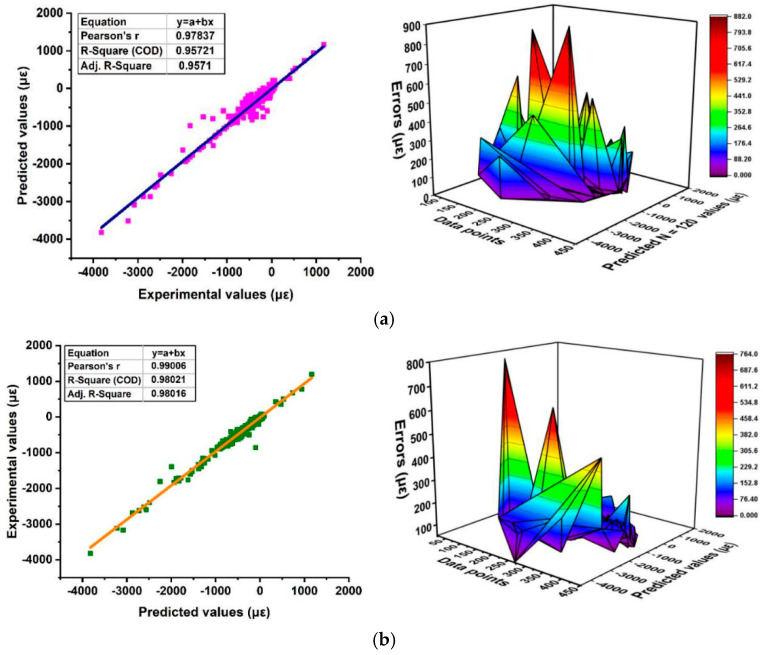
Regression analysis and errors of experimental and predicted values. (**a**) SVR with AdaBoost model, (**b**) RFR model [207].

Qureshi et al. (2022) [207] employed SVR as a standalone ML algorithm, which was then ensemble using boosting and bagging approaches to reduce bias and overfitting. These ensemble techniques were further optimized with 20 sub-models by varying the number of estimators to achieve robust prediction accuracy (R^2^). Additionally, a modified bagging approach using RFR was applied to predict the AS of concrete containing SCMs and SAPs. The dataset included key parameters such as the w/c ratio, w/b ratio, a/c ratio, C, SF, fly ash, BFS, filler, MK, SP, SAP content, SAP size, curing time, and SAP water intake. The performance of the SVR model significantly improved when combined with AdaBoost and modified bagging with RF. The SVR with AdaBoost and RFR models achieved R^2^ values of approximately 0.95 (Figure 10a) and 0.98 (Figure 10b), respectively, showing 17% and 21% improvement over the standalone SVR model, which had an R^2^ of 0.81. Additionally, the modified bagging model demonstrated a 67% improvement in RMSE and a 63% improvement in MAE compared to the SVR model. Similarly, the AdaBoost-enhanced SVR model showed a 47% improvement in RMSE and a 36% improvement in MAE. Overall, both ensemble models delivered strong performance compared to the standalone SVR model. Considering input variables such as w/c, w/b, a/c, C, SF, fly ash, BFS, MK, filler, SP, SAP content, SAP size, SAP water uptake, and time, further modelling was carried out. Four ML models—KNN, RF, GB, and XGB—were evaluated. RF and XGB outperformed the other models, aligning with prior research on cementitious materials. Monte Carlo simulation confirmed XGB’s high accuracy, achieving R^2^ = 0.962 (training) and 0.954 (testing). The predicted values closely matched the experimental shrinkage/expansion measurements, ranging from −3800 µε (shrinkage) to 1200 µε (expansion). XGB models with default hyperparameters proved effective for predicting shrinkage/swelling in SAP-modified concrete. Furthermore, SHAP analysis identified the most influential parameters for shrinkage predictions including the a/c ratio, SAP content, time (days since the start of shrinkage measurements), w/b ratio, cement content, w/c ratio, SAP size, and SF content [208].

#### 3.8.2. Drying Shrinkage (DS)

Several semi-empirical and semi-theoretical models, such as CEB-FIP (1990) [28,29], CEB-FIP (2010) [35], B3, B4, GL 2000, and ACI-209R, have been developed to predict concrete shrinkage based on experimental data and shrinkage theory. However, their results vary significantly due to differences in the experimental backgrounds of each prediction model. In terms of model composition, most prediction models exhibit similar characteristics and can be represented as a combination of three components: the ultimate shrinkage coefficient, the humidity effect coefficient, and the shrinkage development function. While models like CEB-FIP (1990, 2010), GL 2000, and ACI-209R, use power functions to describe the development of drying shrinkage, others, such as B3 and B4, employ hyperbolic tangent functions. However, regardless of the function used, discrepancies remain between the development function and the actual drying shrinkage pattern. This is because the development function serves only as an approximation and cannot fully capture the actual shrinkage behavior [209,210].

Pang et al. (2024) [210] proposed a modified CEB-FIP (1990) [28,29] model to enhance the accuracy of predicting DS in partially encased steel reinforced concrete (PESRC) columns. Initially, a finite element (FE) method was employed to simulate shrinkage deformation in concrete, with results validated against both the original CEB-FIP (1990) [28,29] model and experimental data. This method was subsequently applied to PESRC columns to evaluate how the geometry of the steel section influences moisture diffusion and shrinkage behavior. Based on the simulation outcomes and cross-sectional characteristics, a modified model was developed. The modified CEB-FIP (1990) [28,29] model displayed strong agreement with the FE analysis results and effectively predicted shrinkage in PESRC columns, capturing the differences between reinforced concrete and steel reinforced concrete columns due to moisture diffusion effects.

Zhang et al. (2021) [211] investigated the impact of SAPs on the DS of recycled concrete (RC). natural aggregate was replaced with recycled aggregate at 0%, 50%, and 100%, and RC mixtures with different SAP contents were prepared. Performance was assessed through slump tests, CS tests, and DS tests. The GM (1, N) grey prediction model was used to estimate shrinkage, utilizing shrinkage data from 1, 3, 7, 14, 28, 56, and 90 days as the original dataset. Grey theory is a prediction method that enables forecasting, decision-making, and control despite incomplete information. Results showed that GM (1, N) predictions closely matched actual shrinkage values, with an R^2^ value of 0.90 and a relative error of 0.01, demonstrating the model’s reliability, thereby confirming that grey system theory is effective in predicting DS in SAP-modified RC.

The ANN model was developed incorporating input variables such as W, C, SF, SP, admixture, and age for predicting restrained shrinkage stresses in repair mortar. Three models were developed based on different binder compositions: OPC, 10% SF as a partial cement replacement, and a combination of both. For mortars with OPC, the ANN model achieved an R^2^ of 99.74%, MAE of 0.0808, MAPE of 0.0397, and RMSE of 0.0138. With 10% SF, the values were 99.25%, 0.0090, 0.0731, and 0.3161, respectively. When both binders were combined, the model performed even better, with an R^2^ of 99.77%, MAE of 0.0093, MAPE of 0.0804, and RMSE of 0.1775. Overall, the ANN models significantly outperformed conventional approaches, offering greater accuracy and reliability in predicting restrained shrinkage stresses. These models serve as effective tools for optimizing repair mortar performance, enabling more precise and efficient concrete repair designs [52]. The ANN model, using a backpropagation algorithm for the learning process, considered key factors affecting concrete DS, including RH, curing period, volume-to-surface area ratio, w/c ratio, and sand-to-total aggregate ratio. The ANN model’s outputs were compared with existing models, including ACI, CEB-FIP, GL 2000, B3, ATLANTA, and SB3. The ANN model achieved the highest R value of 0.9834, while the existing models had R values ranging from 0.7842 to 0.9390 [212]. RF was used to predict DS considering variables such as w/c ratio, a/c ratio, C, AEA, 28D CS, volume to surface ratio, environmental humidity of specimen preconditioning, age of loading, specimen height, exposure time for shrinkage, time of measurement, and temperature. During both training and testing, the RF model achieved high reliability, with a R^2^ of 0.89, explaining 89% of the shrinkage variability [213].

Twelve ML algorithms such as DT, KNN, Lasso regressor, Ridge regressor, ElasticNet, linear regression, bagging RF, AdaBoost, XGB, LightGBM, and CatBoost were used to predict DS. The Lasso regressor is designed to enhance prediction accuracy, even for rare features, by minimizing data values. This process reduces data complexity, making it effective for models with numerous input attributes. Additionally, lasso helps prevent overfitting by eliminating less significant features. The Ridge regressor is used to address multicollinearity when independent variables are highly correlated. It reduces the influence of less significant attributes by shrinking their coefficients. In regression analysis, ridge regression helps minimize standard errors by accounting for deviations, improving model stability and accuracy. The ElasticNet Regressor minimizes errors by combining the strengths of Lasso and Ridge regressions. It merges Lasso’s approach of eliminating low-impact features with Ridge’s technique of reducing the coefficients of less significant features, enhancing model performance and stability. The parameters considered for modelling were drying time, replacement ratio, TS, and CS. Among the algorithms tested, DT, XGB, RF, Bagging, and CatBoost achieved a prediction performance exceeding 99%. Among them, CatBoost emerged as the best algorithm with an average R^2^ score of 99.59% [214].

### 3.9. Creep

Creep behavior significantly impacts shrinkage-induced cracking, especially in repair work [215,216]. The prediction of this property has been conducted by researcher using B3 model and various code-based models such as ACI, CEB, European, JSCE, AASHTO LFRD, and more [217,218,219,220]. The B3 model is a reliable method for predicting the long-term deformations of concrete, which is crucial for structural integrity. It takes into account factors such as composition, strength, and water content, using a log-double-power law for creep compliance [30,31]. Unlike code-based models, the B3 model considers the w/c ratio, a factor that other models do not include [218].

Li et al. (2022) [219] investigated the basic tensile creep of early-age concrete with a 0.35 w/b ratio and 0.3% SAP using direct tensile testing. The applicability of the B3 model and MC2010 model [220,221] for predicting early-age tensile creep was assessed, resulting in a modified MC2010 model to improve accuracy. Unlike the B3 model, the MC2010 model represents the creep behavior of cement-based composites using a basic creep coefficient. Both the B3 and MC2010 models underestimated the basic tensile creep of early-age concrete, likely because they were primarily developed based on the compressive creep of mature concrete. To address this limitation, a modified MC2010 model was proposed, incorporating new coefficients. Comparative analysis showed that the revised model better predicts the short-term basic tensile creep of reference concrete, demonstrating good agreement with test data and improved predictive accuracy. However, further research is required to improve and verify the model’s applicability to other concrete mixes containing SAP.

The tensile creep compliance function of HPC internally cured with SAPs was determined using a modified B3 model. This was achieved by comparing the differences in the tensile creep compliance functions of HPC with varying amounts of SAPs and the corresponding equations were derived as follows [222,223]:

(19) $$J(t,t’,\alpha )=J_{0c}(t_{c},t)\;\times 0.68\times \;(1\;-\;1e^{-\;\frac{t-t’}{4.16}})\times (1-0.98\alpha )$$

(20) $$J_{0c}(t_{c},t)=q_{1}+q_{2}\;Q\;(t_{c},t’)+q_{3}\;\ln[1+({t_{c}-t’)}^{0.1}]+q_{4}\;\ln\left(t_{c}/t’\right)$$

(21) $$Q\;(t_{c},t’)=Q_{f}\;(t’)\;{\left[1+{\left(\frac{Q_{f}\;(t’)}{Z(t_{c},t’)}\;\right)}^{r(t’)}\right]}^{-1/r(t’)}$$

(22) $$Q_{f}\;(t’)={\left[0.086\;{\left(t’\right)}^{2/9}+1.21\;{\left(t’\right)}^{4/9}\right]}^{-1}$$

(23) $$Z\;(t_{c},t’)={\left(t’\right)}^{-0.5}\;\ln\left[1+{\left(t-t’\right)}^{0.1}\right]$$

(24) $${r(t’)=1.7(t’)}^{0.12}+8$$

where J(t,t’,α) is the tensile creep compliance function at the equivalent maturity age (*t*) when the amount of SAPs is α. $J_{0c}(t_{c},t)$ is the tensile creep compliance function of the mixture without SAP at the age of cracking. α is the amount of SAP; (*t*) is the equivalent maturity age of HPC while $t_{0}$ is the equivalent maturity age of loading in hours. $t_{c}$ is the equivalent maturity age when the restrained specimen of mixture without SAP cracked. The terms $q_{1}-q_{4}$ are empirical material constitutive parameters derived from concrete strength and composition [222,223]. The tensile creep compliance function values at the cracking age of the restrained SAP-0 specimen, obtained from the above equations or experimental tests, were 85, 71, 55, and 44 με/MPa (from Equations (12)–(17)) or 85, 69, 58, and 43 με/MPa (from tests) for SAP contents of 0%, 0.17%, 0.35%, and 0.49%, respectively. The deviations between the predicted and experimental results were small, ranging from 0% to 5.2%, confirming the reliability of these equations for predicting tensile creep compliance in HPC with SAPs. However, the study did not consider the influence of loading age on early-age basic tensile creep, particularly before three days. Although the proposed model was somewhat arbitrary due to limited data and uncertain physical implications, it effectively captured the mathematical trends observed in the experimental data [222].

Bal and Bodin (2014) [224] utilized an ANN with multilayer backpropagation, trained on a large database of experimental results from the RILEM data bank. The model was developed by considering key parameters such as RH, curing period, w/c ratio, volume-to-surface area ratio (V/S), and fine aggregate-to-total aggregate ratio. The ANN model achieved an R value of 0.9801, validated by comparisons with parametric models like B3 (R = 0.8462), ACI 209 (R = 0.8738), CEB (R = 0.8841), GL 2000 (R = 0.8913), ATLANTA (R = 0.8554), and SB3 (R = 0.8361), demonstrating good agreement in predicting the evolution of creep over time. In another study [225], four ML models—RF, ANN, XGB, and LightGBM (LGBM)—were used to predict UHPC creep behavior. Key features influencing creep were identified, including w/b ratio, a/c ratio, CS at loading age, EM at loading age, loading duration, steel fiber volume content, and curing temperature. Through Bayesian optimization and five-fold cross-validation, the models were fine-tuned, achieving high accuracy (R^2^ = 0.9847, 0.9627, 0.9898, and 0.9933 for RF, ANN, XGB, and LGBM, respectively). SHAP analysis ranked the most influential features for the creep coefficient: loading duration, curing temperature, CS at loading age, and w/b ratio, with results consistent with theoretical understanding.

Ghasemzadeh et al. (2016) [226] developed an Inverse Analysis (IA) method to predict long-term compressive creep in concrete using short-term creep data under consistent conditions. This method was applied to seven established prediction models (including ACI 209R-92 [27], Tadros et al., 2003 [227], Mazloom 2008 [228], Huo et al., 2001 [229], Gilbert 2005 [230], CEB-FIB 1999 [231], and GL 2000 [32,232]) and validated using experimental data from six specimens across three studies. The IA method significantly improved the accuracy of long-term creep predictions, reducing prediction errors to within ±14% for five of the six specimens. Mean square error analysis also confirmed a notable reduction in overall prediction error after applying IA. However, the method’s performance is influenced by the number and precision of short-term measurements—more accurate and abundant data improve results. Additionally, IA is sensitive to models that use logarithmic or hyperbolic time functions. If an effective IA modification is developed for models with these time functions, their adjusted predictions can be averaged with other models, provided they meet Chauvenet’s criterion.

Similarly, another study developed a creep compliance prediction model for concretes containing fly ash and slag as cement substitutes using GPR, ANN, RFR, and Decision Tree Regression (DTR). The RFR and GPR models were the top-performing ML techniques, with R^2^ values of 0.99 for the validation data. The ANN and DTR models also produced accurate results, achieving R^2^ values of 0.91 and 0.90, respectively, for the validation data. Sensitivity analysis of the GPR model showed small variability in performance based on training repetitions, input variable selection, and validation methodology, and the RFR model performed better when the sample size was limited. The SHAP analysis revealed that time was the most influential factor, followed by loading age, CS, EM, volume-to-surface ratio, and RH. Fly ash and SF content had minimal impact on predictions [233].

### 3.10. Permeability and Sulphate Attack Resistance

In the study by Abdelhady et al. (2021) [234], mathematical models proposed in the literature from 1995 to 2021 for predicting the water permeability of PC were collected and classified into five groups based on their mathematical forms. A total of 41 models from 30 studies were evaluated using a constructed database and statistical criteria to identify a consistent model. These models followed various curve trends and were categorized based on their mathematical form and the number of independent variables, including linear, second-degree polynomial, exponential, power, and the Carman–Kozeny relationship. The linear and polynomial models achieved R^2^ values of approximately 0.644 and 0.653, respectively. In contrast, the exponential model had the lowest R^2^ (0.531) among the established models but provided logical predictions. The Carman–Kozeny model also yielded a lower R^2^ (0.546) than the power model. Among all proposed models, the power model was the most effective, achieving the highest R^2^ (0.701) and the lowest values for MAE, RMSE, and Average Absolute Ratio Error (AARE).

Further, the permeability of the cement paste was initially determined using General Effective Media (GEM) theory and subsequently used to calculate the permeability of Engineered Cementitious Composites (ECC) through the General Self-Consistent Scheme (GSCS) method, with most parameters readily obtained from the raw material data. In the GEM theory, cement paste is a two-phase composite: capillary pores with high permeability and a low-permeability phase consisting of C-S-H gel, CH, and unhydrated particles. The GSCS method treats mortar as a three-phase composite of bulk cement paste, spherical sand particles, and the interfacial transition zone (ITZ). Assuming a uniform interface thickness for all sand particles and zero permeability for sand, the matrix mortar’s permeability was determined. The porosity and permeability of the ITZ were first calculated, followed by other parameters derived from mix proportions. The influence of PVA fibers was incorporated based on their volume fraction. Water permeability tests validated the model, showing that PVA fiber addition slightly reduced ECC’s water permeability coefficient. The deviation between calculated and measured permeability coefficients was up to 27.1%, which is within a reasonable range for cement-based materials [235].

Ahmed et al. (2024) [236] examined three predictive modelling techniques, such as LR, NLR, and ANN, to estimate permeability in porous concrete. Using data from 139 samples across multiple studies, with key input parameters such as the w/c ratio, CA, C, porosity, and curing time. In the LR, the results indicated that the w/c ratio and porosity significantly impact the permeability of porous concrete, with the w/c ratio having the greatest effect on increasing permeability. Similarly, in the NLR model, the w/c ratio, C content, and void ratio were found to have a notable influence on permeability. For ANN predictions, the R^2^ value was 0.937 for training data, 0.977 for testing data, and 0.980 for validation data. The findings suggest that ANN outperforms the other models, highlighting its superior predictive capability. Six ML algorithms—LR, ANN, boosted decision tree regression, RFR, KNN, and SVR—were used to predict the permeability of pervious concrete based on mix parameters such as compaction energy, a/c ratio, aggregate size, and ultrasonic velocity. By incorporating non-destructive measurements and mix design variables, these models facilitate practical applications in the construction industry without requiring theoretical expertise. The results show that ANN was the most effective model for predicting porosity (R^2^ = 0.9502 for training and R^2^ = 0.8958 for testing), while boosted decision trees performed best for permeability (R^2^ = 0.9323 for training and R^2^ = 0.7574 for testing). Sensitivity analysis using RFR identified ultrasonic pulse velocity as the most influential factor in predicting permeability [120].

ANN is the most effective model for predicting chloride ion concentration when ample data is available, requiring proper training and parameter tuning for accuracy. DT models offer high interpretability and ease of implementation but may suffer from overfitting and limited accuracy due to tree depth and data fluctuations. SVM models handle nonlinear relationships well and perform effectively with limited data but require careful parameter tuning and can be computationally intensive. Overall, ML methods—including DT, ANN, and SVM—provide flexible and accurate predictions by capturing the nonlinear dynamics of chloride ion diffusion. Advanced techniques like feature selection, regularization, and multi-model integration further improve stability and accuracy, ensuring continued advancements in chloride ion concentration prediction as data availability and algorithms evolve [237].

**Figure 11:**
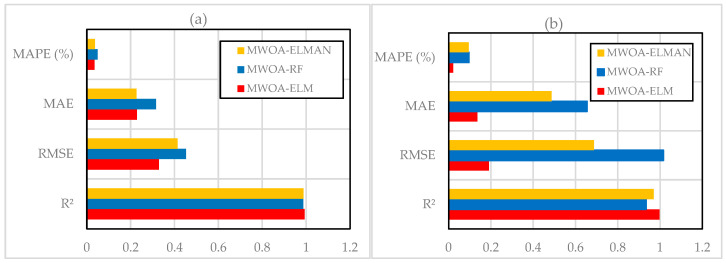
Validation parameters for (**a**) training and (**b**) testing sets for MWOA-ELM, MWOA-RF, and MWOA-ELMAN models [238].

Huang et al. (2023) [238] applied ML models—Extreme Learning Machine (ELM), RF, and Elman Neural Network (ELMAN)—to predict the chloride permeability coefficient of RC, with all three optimized using the Modified Whale Optimization Algorithm (MWOA). The optimized models significantly improved prediction accuracy by 54.4% (ELM), 62.9% (RF), and 36.4% (ELMAN) compared to their initial versions. Among them, the MWOA-ELM model performed best, which can be seen in Figure 11. Comparative analysis showed that traditional models, including MLR (87.15% accuracy), were less effective than the MWOA-ELM model. These results highlight the enhanced predictive capability of ML models optimized with MWOA, offering a superior alternative for chloride permeability coefficient prediction in rubber concrete (RC).

Four ML models—DT, RF, XGB, and LightGBM—were used to assess the resistance of blended cements to external sulphate attack. The models were trained on a dataset incorporating various material and structural properties, including C3S, C2S, C3A, C4AF, C, W, MK, SF, gravel, sand, fly ash, BFS, limestone, w/b ratio, a/c ratio, pH, mold properties, surface/perimeter, and 28D CS. To enhance interpretability, Local Interpretable Model-Agnostic Explanations (LIME) were applied, revealing key patterns in predictions. All models performed well, with LightGBM (R = 0.82) and DT (R = 0.884) achieving the highest accuracy, correctly predicting 80 out of 97 test specimens. Most classification models can rapidly evaluate the sulphate resistance of cementitious materials using the extensive database, while the top regression models can effectively predict the temporal progression of degradation. LIME analysis identified cement proportion, aggregates, and SCM as the most influential factors in determining concrete resistance [239].

Qin et al. (2024) [240] developed a prediction model to estimate failure thickness under sulphate attack, incorporating key factors such as sulphate concentration, initial aluminate content, and sulphate ion diffusion coefficient. The model was based on Fick’s second law and mass conservation principles, assuming a linear relationship between failure thickness and the square root of attack time. The findings indicate that failure thickness increases with higher sulphate concentration and diffusion coefficient but decreases with increased initial aluminate content, showing a negative correlation. The model’s accuracy was validated through comparisons with existing models and experimental data, demonstrating strong alignment within an acceptable error range. This study provides valuable insights for improving durability design and lifespan prediction of concrete structures exposed to sulphate attack.

### 3.11. Model Validation Technique

To evaluate the accuracy of prediction models, several commonly used metrics include the R [66], R^2^ [3,8,9,53,54,62,77,87,119,241], MSE [7,77,78,150], RMSE [3,7,8,9,53,54,62,66,87,119,236,241], MAE [3,7,8,53,54,62,66,87,119,140,236,241], mean absolute percentage error (MAPE) [3,9,12,52,66,148], and normalized mean bias error (NMBE) [12]. These metrics provide quantitative insights into a model’s performance, helping researchers compare and select the most suitable model [7,12]. Ideally, R and R^2^ should be 1 to signify perfect prediction accuracy, while MSE, RMSE, MAE, and MAPE should be 0 for optimal performance. Other error evaluation metrics include root relative squared error (RRSE), root mean squared logarithmic error (RMSLE), normalized mean square error (NMSE), and mean absolute relative error (MARE)—the smaller the values, the smaller the difference between the predicted and measured values [77,78,148,150].

Additionally, several other metrics are used to evaluate performance accuracy. For instance, the 95% Uncertainty (U95) values provide insight into the distribution of predictions relative to the true values, serving as an estimate of prediction dispersion. Higher U95 values indicate greater uncertainty in predictions compared to other models, while lower U95 values suggest a reduced level of uncertainty [62,65]. The overall index (OI) takes into consideration a multitude of performance metrics, with a higher OI value (closer to 1) signifying a well-balanced performance across various metrics [62]. Similarly, the Willmott index (WI) ranges from 0 to 1, with 1 indicating perfect agreement between observed and predicted values. Higher values generally reflect better model performance and are particularly useful for detecting biases that other metrics might overlook. The WI is often compared with R^2^, providing additional insights when R^2^ alone may not fully capture a model’s predictive accuracy [3,242].

Based on the scatter index (SI) value, the proposed model demonstrates poor performance when SI > 0.3, fair performance when 0.2 < SI < 0.3, and excellent performance when SI < 0.1 [3,236]. The mean bias error (MBE) reflects a model’s long-term performance, where a positive MBE indicates underestimation and a negative MBE suggests overestimation. A smaller absolute value signifies better model accuracy [3,243]. The mean absolute deviation (MAD) is computed by averaging the absolute differences between actual and predicted values, helping assess data variability; lower MAD values indicate better model performance [9]. The objective value (OBJ) function is utilized as a critical output parameter to assess the effectiveness of the proposed models [139,236]. The Kling–Gupta efficiency (KGE) combines correlation, bias, and variability components to measure model performance comprehensively, while the Nash–Sutcliffe efficiency (NSE) assesses the relative magnitude of the residual variance compared to the observed data variance, indicating how well the model’s predictions match the observed data [66,77]. The standardization of model evaluation methodologies across studies would facilitate more consistent benchmarking and enable more rigorous comparison of predictive performance.

## 4. Conclusions

A comprehensive review of studies on predicting cementitious material properties using diverse datasets (such as w/c ratio, w/b ratio, and material proportions, etc.), modelling techniques, and validation methods was conducted. Data variability significantly affects modelling accuracy, making proper dataset handling—such as managing missing values, normalization, and outlier detection—crucial prior to model development. While larger datasets generally improve reliability, smaller datasets have also yielded accurate predictions. Standardized, high-quality datasets are essential for improving predictions.

For predicting fresh concrete properties as well as IRH and DOH, ML techniques such as ANN, SVM, RFR, and XGB have demonstrated high efficiency, especially when mix components vary. In addition, mathematical models like YODEL, Herschel–Bulkley, and Krieger–Dougherty effectively predict yield stress and viscosity of fresh concrete.

When predicting CS, traditional models such as Abrams, Slater, and ACI rely heavily on the w/c ratio and often overlook aggregate properties, leading to limited predictive performance. Modified models like Bolomey and Feret improve accuracy by incorporating cement strength but still fall short. Aggregate characteristics have a significant influence on CS, reducing the reliability of these conventional approaches. In contrast, ML techniques, including ensemble and non-ensemble techniques, have demonstrated superior predictive performance. Despite challenges in selecting optimal models and input variables, ML-driven approaches continue to evolve, outperforming conventional numerical models.

Similarly, properties related to durability—such as TS, FS, and EM—are better predicted using ML models. Traditional code-based equations and empirical models often show limitations, whereas ML methods have achieved high accuracy in TS, FS, and EM predictions.

In predicting AS, models like CEB-FIP, EN-1992, and Jonasson and Hedlund tend to underestimate AS, while models like Tazawa and Miyazawa overestimate it. More advanced models, including those by Dilger and Wang and Lee et al., offer improvements but still struggle to fully capture the progression of AS. The B4 model, an enhancement of B3, improves shrinkage assessment by considering concrete composition, while its modified version (B4-mod) further incorporates humidity variations and quicklime effects. On the other hand, ensemble methods and deep learning models prove to be the most effective for shrinkage prediction.

For creep, traditional models like B3 and MC2010 tend to underestimate early-age behavior. Modified versions with updated coefficients have improved performance, particularly for SAP-modified concrete. ML approaches such as ANN, RF, and XGB have shown superior predictive capability. Further studies on properties like permeability and sulphate attack resistance confirm the effectiveness of ML approaches.

Evaluation metrics commonly used for model performance include R, R^2^, MSE, RMSE, MAE, and MAPE. Additional metrics such as RRSE, RMSLE, and NMSE provide deeper insights, while U95 and WI assess prediction uncertainty and agreement. Metrics like SI, MBE, MAD, OBJ, KGE, and NSE further enhance performance evaluation.

Future studies should focus on developing dedicated predictive models that account for various innovative materials—such as internal curing agents—that have gained attention over the past few decades. Incorporating their absorption capacities will be crucial for accurately predicting a wide range of fresh and hardened concrete properties.

The complex interactions among concrete constituents—such as cement, water, and aggregates—are influenced by material properties and environmental conditions, making accurate modelling challenging. Understanding their combined effects on concrete and mortar performance requires carefully developed models. Expanding dataset coverage and refining prediction techniques can enhance robustness and applicability across a wide range of mixtures. ML methods offer superior accuracy, adaptability, and efficiency when analyzing large datasets. However, their performance may vary with changes in input variables or data variability. In contrast, statistical models, though more straightforward to apply, generally yield lower prediction accuracy compared to ML approaches.

## Data Availability

No new data were created or analyzed in this study. Data sharing is not applicable to this article.
